# Awakening a Molecular Mummy: The Inter-and Intramolecular Photochemistry of Pyromellitic Diimides with Alkyl Carboxylates

**DOI:** 10.3390/photochem2030046

**Published:** 2022-08-24

**Authors:** Wolfgang H. Kramer, Donya Razinoubakht, Gurjit Kaur, Axel Klein, Simon Garbe, Jörg Neudörfl, Sabrina Molitor, Anne Zimmer, Axel G. Griesbeck

**Affiliations:** 1 Department of Chemistry and Biochemistry, Millsaps College, 1701 North State Street, Jackson, MS 39210, USA; 2 Department of Chemistry, Faculty of Mathematics and Natural Sciences, University of Cologne, Inorganic Chemistry, Greinstr. 6, 50939 Köln, Germany; 3 Department of Chemistry, Faculty of Mathematics and Natural Sciences, University of Cologne, Organic Chemistry, Greinstr. 4, 50939 Köln, Germany

**Keywords:** pyromellitic imides, photodecarboxylation, electron transfer, radical ions, alkylative degradation

## Abstract

Pyromellitic acid diimides are not as chemically unreactive as conjecturable (and presupposed) from their numerous applications as electron acceptor units or electron carriers in molecular donor–acceptor dyads or triads. Similar to the corresponding phthalimides, electronically excited pyromellitic diimides oxidize alkyl carboxylates in aqueous solution via intermolecular electron transfer (PET) processes, which eventually results in radical–radical combination products, e.g., the benzylation product **6** from *N,N*′-dimethyl pyromellitic diimide **5**. The analogous product **7** was formed with pivalic acid as *tert*-butyl radical source. One additional product **8** was isolated from alkylation/dearomatization and multiple radical additions, respectively, after prolonged irradiation. In intramolecular versions, from *N*-carboxyalkylated pyromellitic diimides **9a–e** (C_1_ to C_5_-spaced), degradation processes were detected, e.g., the cyclization products **10** from the GABA substrate **9c**. In sharp contrast to phthalimide photochemistry, the green pyromellitic diimide radical anion was detected here by UV-vis absorption *(λ*_abs_ = 720 nm), EPR (from **9d**), and NMR spectroscopy for several intramolecular electron transfer examples. Only the yellow 1,4-quinodial structure is formed from intermolecular PET, which was deduced from the absorption spectra (*λ*_abs_ = 440 nm) and the subsequent chemistry. The pyromellitimide radical anion lives for hours at room temperature in the dark, but is further degraded under photochemical reaction conditions.

## 1. Introduction

**Figure 1.**
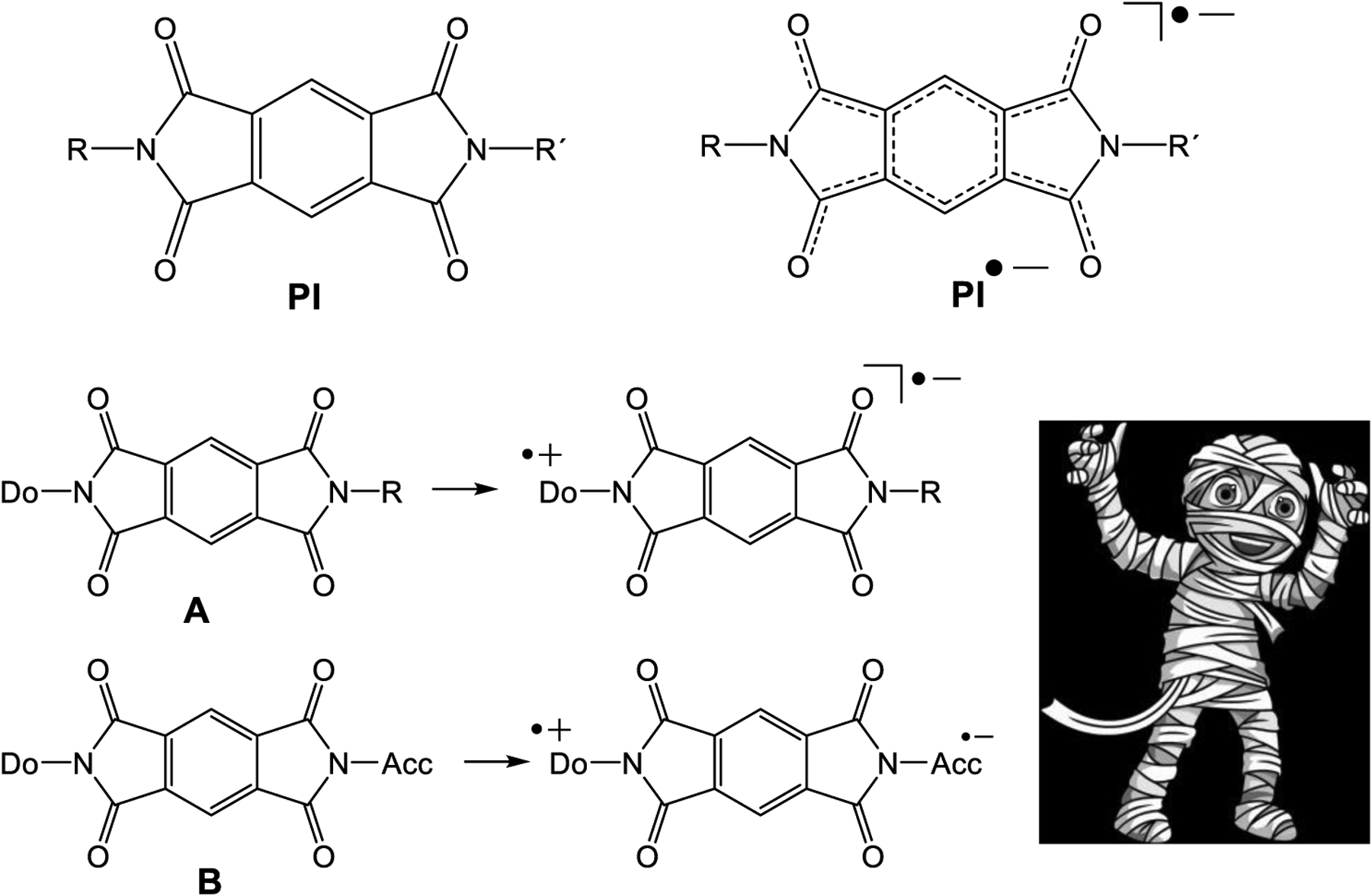
The pyromellitic acid diimide (PI) and the PI anion radical (PI^•−^). A: PI with donor in a dyad. B: PI with donor and acceptor acts as a molecular wire and chemically stable molecular mummy.

Numerous applications of pyromellitic acid diimides (pyromellitic imides, **PI**, Figure 1) as electron acceptors in dyads with N-linked electron donors (**A**) [1–4] or as electron carriers in donor–acceptor substituted model compounds that mimic the photosynthetic reaction center (**B**) have been described [5–12]. Plenty of applications were developed that harvest excitation energies in electron transfer/carrier systems. In many of these often complex and high-molecular-weight compounds, **PI** operate as reliable and chemically stable electron carriers, a kind of molecular mummy [1–12]. At least, this is the message of numerous publications and the basic requirement for all applications.

**Scheme 1.**
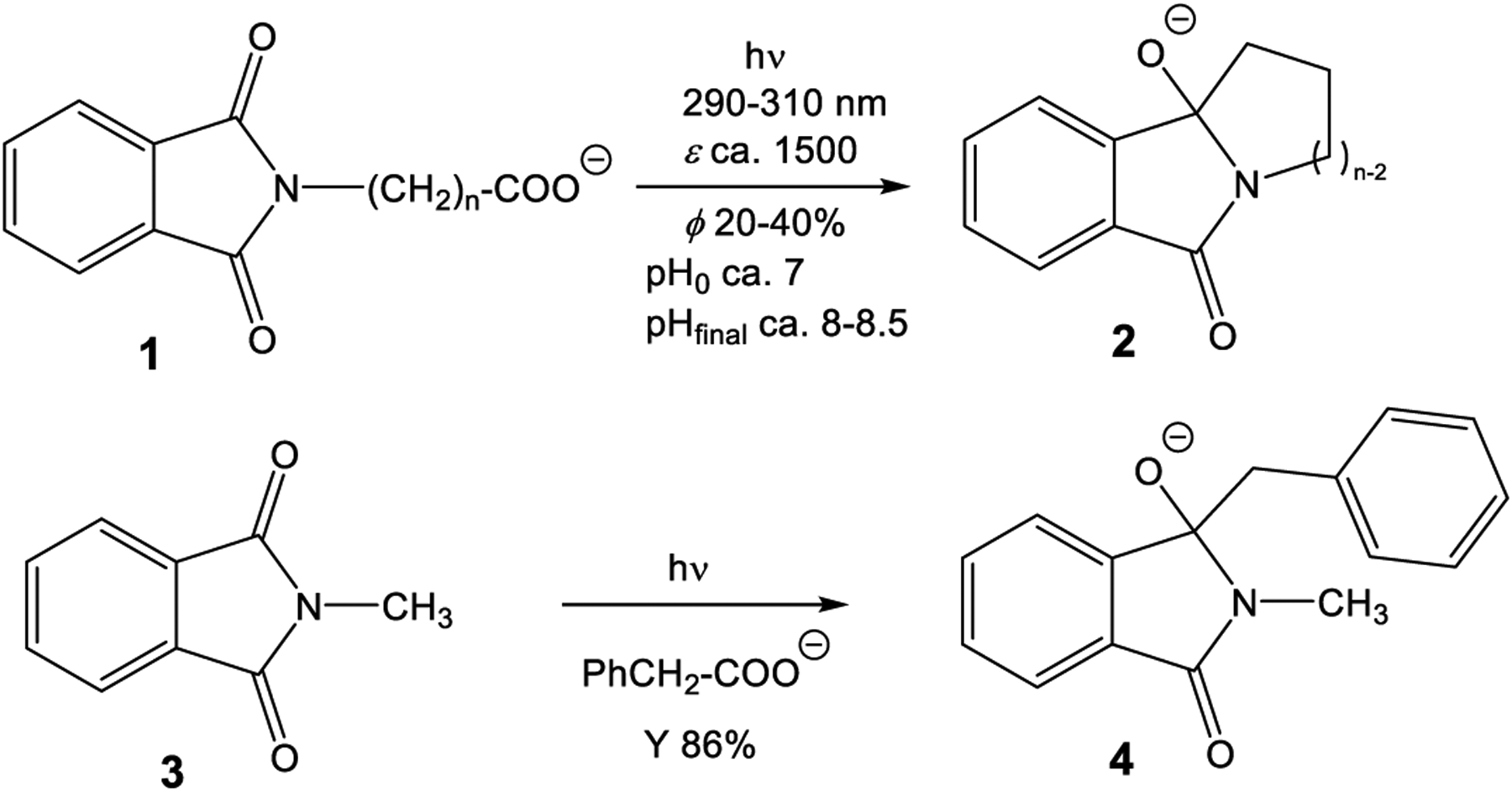
Intra- and intermolecular photoredox decarboxylative cyclization/addition of phthalimides.

Additionally and more importantly, pyromellitimide is the central building block of a huge number of polymers that exhibit remarkable mechanical and thermal stability properties. One well-known example is the oxydiphenylene–pyromellitimide polymer Kapton (poly(4,4’-oxydiphenylene-pyromellitimide)) [13], also described as a radiation-stable material that allows applications in spaceflights, such as coatings of satellites [14,15]. Apparently, the radical anion of pyromellitimides (**PI**^•−^) is either too short-lived or too unreactive to lead to chemical reactions, e.g., decomposition with loss of π-conjugation. At first sight, this behavior reported in the literature is however inconsistent with our experience that we gathered from phthalimide photochemistry in the last decade [16–21]. These electron-accepting compounds are susceptible to radical coupling reactions following electronic excitation of the (mono)imide chromophore. These processes allow the formation of ring-annulated isoindolinones **2** from the corresponding carboxylates 1 in high chemical yields and good quantum yields by irradiation in the UV-A of the phthalimide chromophore and initiated by a photoinduced electron transfer (PET) followed by decarboxylation and radical-radical coupling (Scheme 1). Overall, the negative charge is carried from carboxylate to alkoxide and thus the reaction can be followed by pH and conductivity measurements [22].

These radical coupling reactions are possible not only in intramolecular fashion but also intermolecular radical addition processes can be designed for numerous phthalimide electron acceptors (e.g., the *N*-methyl compound **3** in Scheme 1). Well-suited electron donors for PET and rapid decarboxylation are aryl acetates that we have intensively studied and lead to benzylated products **4** [23–29]. These reactions deliver benzylated hydroxyisoindolinones, valuable precursors to numerous pharmaceutically relevant products, such as isoindolinones or isoindoles [30,31]. It is remarkable that these radical combinations efficiently work also with unstabilized radicals from the corresponding carboxylates, e.g., methylation from the PET-oxidation of acetate or H-transfer using formate as the hydrogen atom precursor [32].

While pyromellitimides do not provide this remarkable potential for synthetic applications, their extensive use in donor–acceptor systems due to their favorable redox (*E* = −0.71 eV [33]) and spectroscopic properties (*λ*_max_(PI^*•*−^) = 718 nm [33]) poses the question of whether photoreactions analogous to the ones observed for phthalimides are taking place. In a figurative sense, pyromellitimides and other polyaromatic acceptors are often characterized as highly chemically stable and able to undergo electron transfer cycles for numerous times without chemical degradation. We herein describe the transfer of the well-studied phthalimide electron transfer photochemistry on pyromellitimides. The reported high stability of this chromophore should contradict analogous radical-type reactivity. This, however, does not seem to be true.

## 2. Materials and Methods

### 2.1. Chemicals and Solvents for Syntheses and Photoreactions

Chemicals were purchased from Acros and Fisher and utilized as received. Solvents were purchased from usual vendors and distilled before use.

### 2.2. Synthesis of the Photochemical Starting Materials

*N,N*-dimethyl pyromellitic diimide **5** was prepared according to [34] (CAS 26011-79-0). General procedure for the synthesis of ω-carboxy pyromellitic diimides [35–37]:

One equivalent of pyromellitic anhydride was combined with 2.2 equivalents of desired amino acid in approximately 30 to 40 mL DMF (for about 5 g of anhydride) in a round-bottomed flask. The solution was stirred and heated to approximately 150 °C for 2 h, precipitated with H_2_O, recrystallized from acetone/H_2_O, vacuum-filtered, and the product air-dried.

2,2’-(1,3,5,7-tetraoxo-5,7-dihydropyrrolo[3,4-*f*]isoindole-2,6(1*H*,3*H*)-diyl)diacetic acid (**9a**, n = 1, CAS 7561-08-2)

Yield: 69%; ^1^H NMR (500 MHz, DMSO-*d*_6_): *δ* (ppm) = 12.01 (bs, 2H, COOH), 8.34 (s, 2 H_arom_), 4.38 (s, 4 H, CH_2_); ^13^C NMR (125 MHz, DMSO-*d*_6_): *δ* (ppm) = 169.0 (s, 2 C, COOH), 165.9 (s, 4 C, CON), 137.4 (s, 4 C, Cq), 118.7 (d, 2 C, CH), 39.8 (t, 2 C, CH_2_).

3,3’-(1,3,5,7-tetraoxo-5,7-dihydropyrrolo[3,4-*f*]isoindole-2,6(1*H*,3*H*)-diyl)dipropionic acid (**9b**, n = 2)

Yield: 79%; ^1^H NMR (500 MHz, DMSO-*d*_6_): *δ* (ppm) = 11.99 (bs, 2H, COOH), 8.19 (s, 2 H, H_arom_), 3.82 (t, *J* = 7.3 Hz, 4 H, NCH_2_), 2.63 (t, *J* = 7.3 Hz, 4 H, CH_2_); ^13^C NMR (125 MHz, DMSO-*d*_6_): *δ* (ppm) = 172.5 (s, 2 C, COOH), 166.5 (s, 4 C, CON), 137.4 (s, 4 C, Cq), 117.7 (d, 2 C, CH), 34.5 (t, 2 C, NCH_2_), 32.6 (t, 2 C, CH_2_).

4,4’-(1,3,5,7-tetraoxo-5,7-dihydropyrrolo[3,4-*f*]isoindole-2,6(1*H*,3*H*)-diyl)dibutyric acid (**9c**, n = 3, CAS 61052-99-1)

Yield: 85%; ^1^H NMR (500 MHz, DMSO-*d*_6_): *δ* (ppm) = 12.05 (bs, 2H, COOH), 8.13 (s, 2 H, H_arom_), 3.64 (t, *J* = 6.7 Hz, 4 H, NCH_2_), 2.28 (t, *J* = 7.3 Hz, 4 H, CH_2_COO), 1.90–1.81 (m, 4 H, CH_2_); ^13^C NMR (125 MHz, DMSO-*d*_6_): *δ* (ppm) = 174.3 (s, 2 C, COOH), 166.8 (s, 4 C, CON), 137.4 (s, 4 C, Cq), 117.5 (d, 2 C, CH), 37.9 (t, 2 C, NCH_2_), 31.4 (t, 2 C, CH_2_COO), 23.6 (t, 2 C, CH_2_).

5,5’-(1,3,5,7-tetraoxo-5,7-dihydropyrrolo[3,4-*f*]isoindole-2,6(1*H*,3*H*)-diyl)dipentanoic acid (**9d**, n = 4)

Yield: 82%; ^1^H NMR (500 MHz, DMSO-*d*_6_): *δ* (ppm) = 11.99 (bs, 2H, COOH), 8.15 (s, 2 H, H_arom_), 3.60 (t, *J* = 6.6 Hz, 4 H, NCH_2_), 2.22 (t, *J* = 7.1 Hz, 4 H, CH_2_COO), 1.68–1.59 (m, 4 H, CH_2_), 1.56–1.47 (m, 4 H, CH_2_); ^13^C NMR (125 MHz, DMSO-*d*_6_): *δ* (ppm) = 174.7 (s, 2 C, COOH), 166.8 (s, 4 C, CON), 137.4 (s, 4 C, Cq), 117.6 (d, 2 C, CH), 38.1 (t, 2 C, NCH_2_), 33.5 (t, 2 C, CH_2_COO), 27.8 (t, 2 C, CH_2_), 22.2 (t, 2 C, CH_2_).

6,6’-(1,3,5,7-tetraoxo-5,7-dihydropyrrolo[3,4-*f*]isoindole-2,6(1*H*,3*H*)-diyl)dihexanoic acid (**9e**, n = 5)

Yield: 85%; ^1^H NMR (500 MHz, DMSO-*d*_6_): *δ* (ppm) = 11.97 (bs, 2H, COOH), 8.16 (s, 2 H, H_arom_), 3.61 (t, *J* = 7.0 Hz, 4 H, NCH_2_), 2.19 (t, *J* = 7.2 Hz, 4 H, CH_2_COO), 1.62–1.67 (m, 4 H, CH_2_), 1.57–1.47 (m, 4 H, CH_2_), 1.35–1.25 (m, 4 H, CH_2_); ^13^C NMR (125 MHz, DMSO-*d*_6_): *δ* (ppm) = 174.8 (s, 2 C, COOH), 166.8 (s, 4 C, CON), 137.4 (s, 4 C, Cq), 117.6 (d, 2 C, CH), 38.3 (t, 2 C, NCH_2_), 33.9 (t, 2 C, CH_2_COO), 28.0 (t, 2 C, CH_2_), 26.2 (t, 2 C, CH_2_), 24.5 (t, 2 C, CH_2_).

### 2.3. Photoreactions

Typical photoreaction for the intermolecular photoreaction of **5** with carboxylates:

2 mmol of the corresponding carboxylate were dissolved in 90 mL of water. After addition of 270 mL of acetone, 1 mmol of **5** was added. The solution was then flushed with argon for 5 min and irradiated at 300 nm for 2 h. After the photoreaction, the solution was extracted 3 times with CH_2_Cl_2_. The combined organic phases were washed with 5%-NaHCO_3_ solution and brine. The solution was dried over MgSO_4_, filtered and evaporated under vacuum. The products were purified by column chromatography or recrystallization.

7-benzyl-7-hydroxy-2,6-dimethyl-6,7-dihydropyrrolo[3,4-*f*]isoindole-1,3,5(2*H*)-trione (**6**) (see Supplemental Material S3)

Yield: 56% after recrystallization from chloroform; M: [C_19_H_16_N_2_O_4_]: 336 g/mol; *R*_F_-value: 0.19 (CH_2_Cl_2_/acetone 9:1); Mp.: 273 °C; ^1^H NMR (500 MHz, DMSO-*d*_6_): *δ* (ppm): 8.05 (d, *J* = 0.8 Hz, 1H, 10-H), 7.72 (d, *J* = 0.8 Hz, 1H, 4-H), 7.08–7.04 (m, 3H, 16-H, 17-H), 6.88–6.84 (m, 2H, 15-H), 6.85 (s, 1H, OH), 3.59 (d, *J* = 13.9 Hz, 1H, 13-H), 3.32 (d, *J* = 13.9 Hz, 1H, 13-H), 3.06 (s, 3H, 7-H), 3.04 (s, 3H, 1-H); ^13^C NMR (125 MHz, DMSO-*d*_6_): *δ* (ppm): 167.2 (s, C-8), 166.9 (s, C-6), 165.0 (s, C-2), 153.2 (s, C-11), 136.6 (s, C-3), 134.7 (s, C-9), 134.6 (s, C-14), 132.9 (s, C-5), 129.7 (d, C-15), 127.7 (d, C-16), 126.5 (d, C-17), 117.9 (d, C-10), 116.0 (d, C-4), 90.2 (s, C-12), 41.2 (t, C-13), 23.9 (q, C-7), 23.9 (q, C-1); IR (ATR): ῦ (cm^−1^): 3452 (w), 1765 (w), 1707 (s), 1684 (m), 1425 (w), 1407 (w), 1382 (m), 1071 (w), 1051 (w), 1020 (m), 992 (w), 781 (w), 744 (m), 702 (s), 688 (w); GC/MS (EI, 70 eV): *τ*_R_ = 18.44 min *m/z* (%) = 318 (M^+^-H_2_O, 100), 289 (25), 232 (38), 204 (64), 190 (39), 177 (48), 163 (39), 116 (34), 91 (28), 89 (37), 74 (31). HR-MS (ESI): calcd. for [M + H]^+^ 337.1182835 amu, found 337.11871 amu; calcd. for [M + Na]^+^ 359.1002282 amu, found 359.10085 amu; UV-vis: (MeCN, *c* = 10^−4^ mol L^−1^) *λ*_max_ = 296 nm (*ε* = 2250 L mol^−1^ cm^−1^); Fluorescence (MeCN, *c* = 10^−4^ mol L^−1^, *λ*_ex_ = 300 nm) *λ*_em_ = 453, 346, 308 nm.

7-(*tert*-butyl)-7-hydroxy-2,6-dimethyl-6,7-dihydropyrrolo[3,4-*f*]isoindole-1,3,5(2*H*)-trione (**7**) (CCDC 2191095, see Supplemental Material S4)

Yield: 33 %; M: [C_16_H_18_N_2_O_4_]: 302 g/mol, *R*_F_-Value: 0.23 (CH_2_Cl_2_/acetone 9:1); Mp.: 248 °C; ^1^H NMR (500 MHz, DMSO-*d*_6_): *δ* (ppm): 7.93 (d, *J* = 0.4 Hz, 1H, 10-H), 7.90 (d, *J* = 0.8 Hz, 1H, 4-H), 6.77 (s, 1H, OH), 3.07 (s, 3H, 7-H), 2.99 (s, 3H, 1-H), 0.98 (s, 9H, 14-H); ^13^C NMR (125 MHz, DMSO-*d*_6_): *δ* (ppm): 167.3 (s, C-8), 167.0 (s, C-6), 164.9 (s, C-2), 153.9 (s, C-11), 137.5 (s, C-3), 134.2 (s, C-9), 132.9 (s, C-5), 118.4 (d, C-10), 116.1 (d, C-4), 93.9 (s, C-12), 39.4 (s, C-13), 27.5 (q, C-7), 25.9 (q, C-14), 24.0 (q, C-1); IR (ATR): ῦ (cm^−1^): 3338 (w), 1774 (w), 1721 (s), 1674 (s), 1480 (w), 1422 (m), 1380 (s), 1223 (w), 1134 (m), 1026 (s), 1016 (m), 986 (w), 941 (w), 740 (s), 692 (w); HR-MS (ESI): calcd. for [M + H]^+^ 303.1339336 amu, found 303.13435 amu, calcd. for [M + Na]^+^ 325.1158782 amu, found 325.11628 amu, UV-vis: (MeCN, *c* = 10^− 4^ mol L^−1^) *λ*_max_ = 298 nm (*ε* = 2660 L mol^−1^ cm^−1^). Fluorescence: (MeCN, *c* = 10^−4^ mol L^−1^, *λ*_ex_ = 300 nm) *λ*_em_ = 464, 348, 308 nm.

3a,7a-di-*tert*-butyl-2,6-dimethyl-4-(2-oxopropyl)-3a,4,4a,7a-tetrahydropyrrolo [3,4-*f*] isoindole-1,3,5,7(2*H*,6*H*)-tetraone (**8**) (CCDC 1439632)

Yield: 24 %; M: [C_23_H_32_N_2_O_5_]: 417 g/mol; ^1^H NMR (300 MHz, CDCl_3_): *δ* (ppm): 7.14 (s, 1H, 10-H), 3.28 (m, 1H, 4-H), 3.02 (d, *J* = 4 Hz, 1H, 17-H), 2.95 (s, 3H, 1-H), 2.92 (d, *J* = 3.1 Hz, 1H, 17-H), 2.78 (s, 3H, 7-H), 2.18 (s, 3H, 19-H), 0.98 (s, 9H, 14-H), 0.78 (s, 9H, H-16); ^13^C NMR (75.5 MHz, CDCl_3_): *δ* (ppm): 207.4 (s, C-18), 178.9 (s, C-8), 177.0 (s, C-2), 176.9 (s, C-6), 168.9 (s, C-12), 135.5 (s, C-11), 131.2 (d, C-10), 55.6 (s, C-3), 54.2 (s, C-9), 47.3 (d, C-5), 47.3 (t, C-17), 39.9 (s, C-15), 39.3 (s, C-13), 31.9 (d, C-4), 29.9 (q, C-19), 27.0 (q, C16), 25.8 (q, C-14), 24.7 (q, C-7), 23.9 (q, C-1); GC/MS (EI, 70 eV): *τ*_R_ = 16.58 min, *m/z* (%) = 360 (M^+^-acetone, 18), 303 (58), 246 (65), 245 (100).

Typical photoreaction for the intramolecular substrates **9a**–**e**:

About 2.20 mmol of pyromellitic diimide *ω*-carboxylic acid was dissolved in about 10 mL of acetone. An equimolar amount (2.20 mmol) of K_2_CO_3_ was dissolved in 10 mL of water and the two solutions were mixed and stirred until completely clear and no more gas evolved. After addition of 230 mL of water, the solution was flushed with argon for 5 min and then irradiated at 300 nm for 12 h. After the photoreaction, the solution was extracted 3 times with ethyl acetate. The combined organic phases were dried over MgSO_4_, filtered and evaporated under vacuum. This fraction contained the monocyclic products. The aqueous phase was then brought to pH 3 with conc. HCl, and again extracted three times with ethyl acetate. Those combined organic phases were dried over magnesium sulfate, filtered, and evaporated under vacuum. This second fraction contained the bis-cyclic products.

4-(9a-hydroxy-1,3,5-trioxo-1,5,7,8,9,9a-hexahydrodipyrrolo[2,1-*a*:3’,4’-*f*]isoindole-2(3*H*)-yl)butanoic acid (**10c**) (see Supplemental Material S5)

Yield: 24.4 %;M: [C_17_H_16_N_2_O_6_]: 344.32g/mol; ^1^H NMR (300 MHz, DMSO-*d*_6_): *δ* (ppm): 8.01 (s, 1 H), 7.86 (s, 1 H), 3.63 (t, *J* = 6.6 Hz, 2 H, NCH_2_), 3.32–3.59 (m, 2 H), 2.40–2.64 (m, 1 H), 2.28 (t, *J* = 6.6 Hz, 2 H, CH_2_COO), 2.16–2.36 (m, 2 H), 1.85–1.81 (m, 2 H), 1.52 (dd, *J* = 10.5, 10.8 Hz, 1 H); ^13^C NMR (75.5 MHz, DMSO-*d*_6_): *δ* (ppm): 174.3 (s, 1 C, COOH), 167.6 (s, 1 C, CON), 167.3 (s, 1 C, CON), 154.5 (s, 1 C, CON), 137.4 (s, 1 C, Cq), 137.2 (s, 1 C, Cq), 136.0 (s, 1 C, Cq), 133.5 (s, 1 C, Cq), 118.2 (d, 1 C, CH), 117.6 (d, 1 C, CH), 95.8 (s, 1 C, COH), 41.9 (t, 1 C, NCH_2_), 37.7 (t, 1 C, NCH_2_), 35.7 (t, 1 C, CH_2_), 31.4 (t, 1 C, CH_2_COO), 27.7 (t, 1 C, CH_2_), 23.7 (t, 1 C, CH_2_).

5-(10a-hydroxy-1,3,5-trioxo-5,7,8,9,10,10a-hexahydro-1H-pyrido[2,1-*a*]pyrrolo[3,4-*f*]isoindol-2(3*H*)-yl)pentanoic acid (**10d**) (see Supplemental Material S6)

Yield: 25%; M: [C_19_H_20_N_2_O_6_]: 372.38g/mol; ^1^H NMR (500 MHz, DMSO-*d*_6_): *δ* (ppm): 8.03 (s, 1 H), 7.92 (s, 1 H), 4.04 (dd, *J* = 4.5, 12.5 Hz, 1 H), 3.59 (t, 2 H, NCH_2_), 3.73–3.68 (m, 1 H), 3.24–3.17 and 3.68–3.74 (m, 1 H), 3.04 (dt, *J* = 3, 13 Hz, 1 H), 2.37–2.48 (m, 1 H), 2.22 (t, *J* = 7.5 Hz, 2 H, CH_2_COO), 1. 88–1.92 (m, 1 H), 1.66–1.71 (m, 1 H), 1.57–1.66 (m, 2 H), 1.45–1.54 (m, 2 H), 1.26 (dt, *J* = 4, 13.5 Hz, 1 H); ^13^C NMR (125 MHz, DMSO-*d*_6_): *δ* (ppm): 174.6 (s, 1 C, COOH), 167.6 (s, 1 C, CON), 167.4 (s, 1 C, CON), 155.6 (s, 1 C, CON), 136.7 (s, 1 C, Cq), 135.4 (s, 1 C, Cq), 134.9 (s, 1 C, Cq), 133.3 (s, 1 C, Cq), 117.5 (d, 1 C, CH), 117.3 (d, 1 C, CH), 85.7 (s, 1 C, COH), 37.9 (t, 1 C, NCH_2_), 36.5 (t, 1 C, NCH_2_), 35.3 (t, 1 C, CH_2_), 33.5 (t, 1 C, CH_2_COO), 27.8 (t, 1 C, CH_2_), 24.9 (t, 1 C, CH_2_), 22.2 (t, 1 C, CH_2_), 19.5 (t, 1 C, CH_2_).

6-(11a-hydroxy-1,3,5-trioxo-1,5,7,8,9,10,11,11a-octahydroazepino[2,1-*a*]pyrroloisoindol-2(3H)-yl)hexanoic acid (**10e**) (see Supplemental Material S7)

Yield: 61%; M: [C_21_H_24_N_2_O_6_]: 400.43 g/mol; ^1^H NMR (500 MHz, DMSO-*d*_6_): *δ* (ppm): 8.03 (s, 1 H), 7.83 (s, 1 H), 3.59 (t, 2 H, NCH_2_), 3.22 (m, 2 H, NCH_2_), 2.15–2.25 (m, 4 H), 1.43–1.68 (m, 8 H), 1.21–1.39 (m, 4 H); ^13^C NMR (125 MHz, DMSO-*d*_6_): *δ* (ppm): 174.8 (s, 1 C, COOH), 167.4 (s, 1 C, CON), 167.2 (s, 1 C, CON), 143.5 (s, 1 C, CON),), 137.6 (s, 1 C, Cq), 133.6 (s, 1 C, Cq), 132.5 (s, 1 C, Cq), 133.3 (s, 1 C, Cq), 123.5 (d, 1 C, CH), 122.5 (d, 1 C, CH), 90.6 (s, 1C, COH), 39.5 (t, 1 C, NCH_2_), 38.0 (t, 1 C, NCH_2_), 34.0 (t, 1 C,), 33.9 (t, 1 C, CH_2_COO), 28.9 (t, 1 C, CH_2_), 28.0 (t, 1 C, CH_2_), 26.4 (t, 1 C, CH_2_), 26.2 (t, 1 C, CH_2_), 24.7 (t, 1 C, CH_2_), 24.5 (t, 1 C, CH_2_).

11a,12b-dihydroxy-1,2,3,9,10,11,11a,12b-octahydro-5H,7H-pyrrolo[2,1-*a*]pyrrolizino [2,1-*f*]isoindole-5,7-dione (**11c-cis**) and 6b,12b-dihydroxy-1,2,3,6b,7,8,9,12b-octahydro pyrrolo[2,1-*a*]pyrrolizino[1,2-*f*]isoindole-5,11-dione (**11c-trans**)

Yield: 6%; M: [C_16_H_16_N_2_O_4_]: 300.31g/mol, could not be separated; ^13^C NMR (125 MHz, DMSO-d_6_) of 90 ppm region see Supplemental Material S8.

13a,14b-dihydroxy-1,3,4,11,12,13,13a,14b-octahydro-*2H,6H*-indolizino[2,1-*f*]pyrido[2,1-*a*] isoindole-6,8(10*H*)-dione (**11d-cis**) and 7b,14b-dihydroxy-1,2,3,4,7b,8,9,10, 11,14b-decahydro-*6H,13H*-indolizino[1,2-*f*]pyrido[2,1-*a*]isoindole-6,13-dione (**11d-trans**)

Yield: 22%; M: [C_18_H_20_N_2_O_4_]: 328.37 g/mol, could not be separated; ^13^C NMR (125 MHz, DMSO-d_6_) of 90 ppm region see Supplemental Material S9.

15a,16b-dihydroxy-1,2,3,4,5,11,12,13,14,15,15a,16b-dodecahydro-*7H,9H*-azepino[2,1-*a*] azepino[1’,2’:1,5]pyrrolo[3,4-*f*]isoindole-7,9-dione (**11e-cis**) and 8b,16b-dihydroxy- 1,2,3,4,5, 8b,9,10,11,12,13,16b-dodecahydroazepino[2,1-*a*]azepino[1’,2’:1,2]pyrrolo[3,4-*f*]isoindole-7, 15-dione (**11e-trans**)

Yield: 15%; M: [C_20_H_24_N_2_O_4_]: 356.42 g/mol, could not be separated; ^13^C NMR (125 MHz, DMSO-d_6_) of 90 ppm region see Supplemental Material S10.

2,3,9,10-tetrahydro-*5H,7H*-pyrrolo[2,1-a]pyrrolizino[2,1-*f*]isoindolin-5,7-dione (**12c-cis**)

160 mg (0.5 mmol) of the product mixture of **11c-trans** and **11c-cis** was suspended in 25 mL of CH_2_Cl_2_ and a few drops of TFA were added while stirring, until a clear solution resulted. After stirring for 10 min, the solution was washed with 5% bicarbonate solution and brine. After drying over MgSO_4_, the solvent was removed and column chromatography (silica gel, CH_2_Cl_2_/MeOH 100:1) yielded two products as yellow solids. One of them could be identified as **12c-cis**. The other product could not be analyzed by NMR due to solubility issues. Its HR-MS spectrum confirmed the expected mass for **12c-trans**.

**12c-cis:** ^1^H NMR (300 MHz, CD_3_COOD): *δ* (ppm) = 8.02 (s, 1H, H 4), 7.98 (s, 1H, H 3), 6.35 (t, *J* = 3.0 Hz, 2H, H-6), 4.12 (t, *J* = 7.3 Hz, 4H, H 8), 3.45 (m, 4H, H 7); ^13^C NMR (75.5 MHz, CD_3_COOD): *δ* (ppm) = 164.2 (s, C 9), 142.0 (s, C 5), 137.7 (s, C 2), 133.9 (s, C 1), 119.7 (d, C 3), 117.4 (d, C 4), 114.5 (d, C 6), 42.3 (t, C 8), 37.0 (t, C 7). HR-MS (ESI): calcd. For [M + H^+^] 265.0971542, found 265.09744 amu, calcd. for [M + Na^+^] 287.0790989, found 287.07949 amu; other fraction, presumably **12c-trans**: HR-MS (ESI) calcd. For [M + H^+^] 265.0971542, found 265.09746 amu, calcd. for [M + Na^+^] 287.0790989, found 287.07944 amu.

## 3. Results

### 3.1. Intermolecular Reactivity

**Figure 2.**
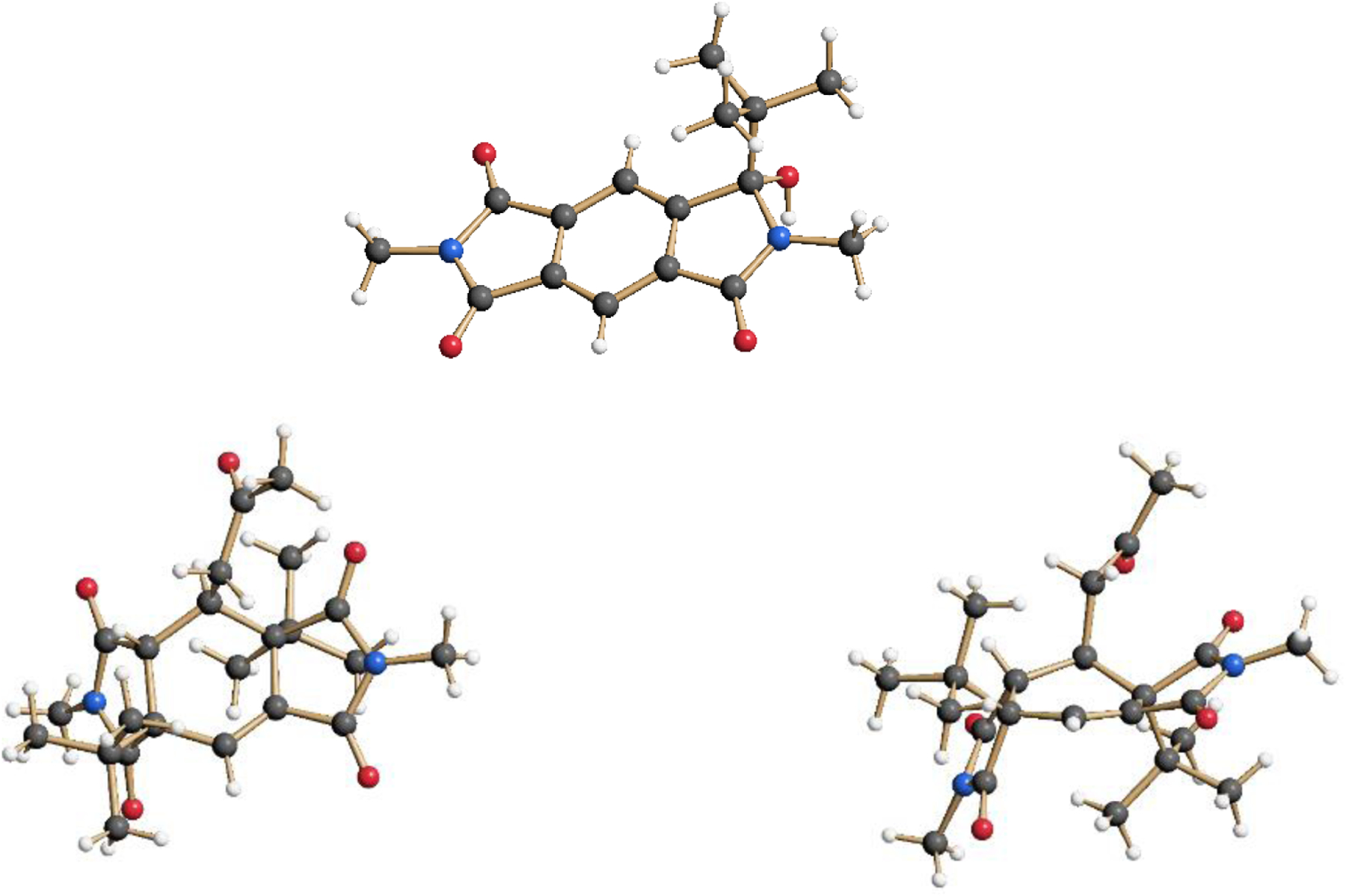
Monoaddition product **7** and the double-addition and solvent-trapping product **8** in the crystal. Compound **8** is shown from two views in the crystal structure (top and side view on the central ring).

**Scheme 2.**
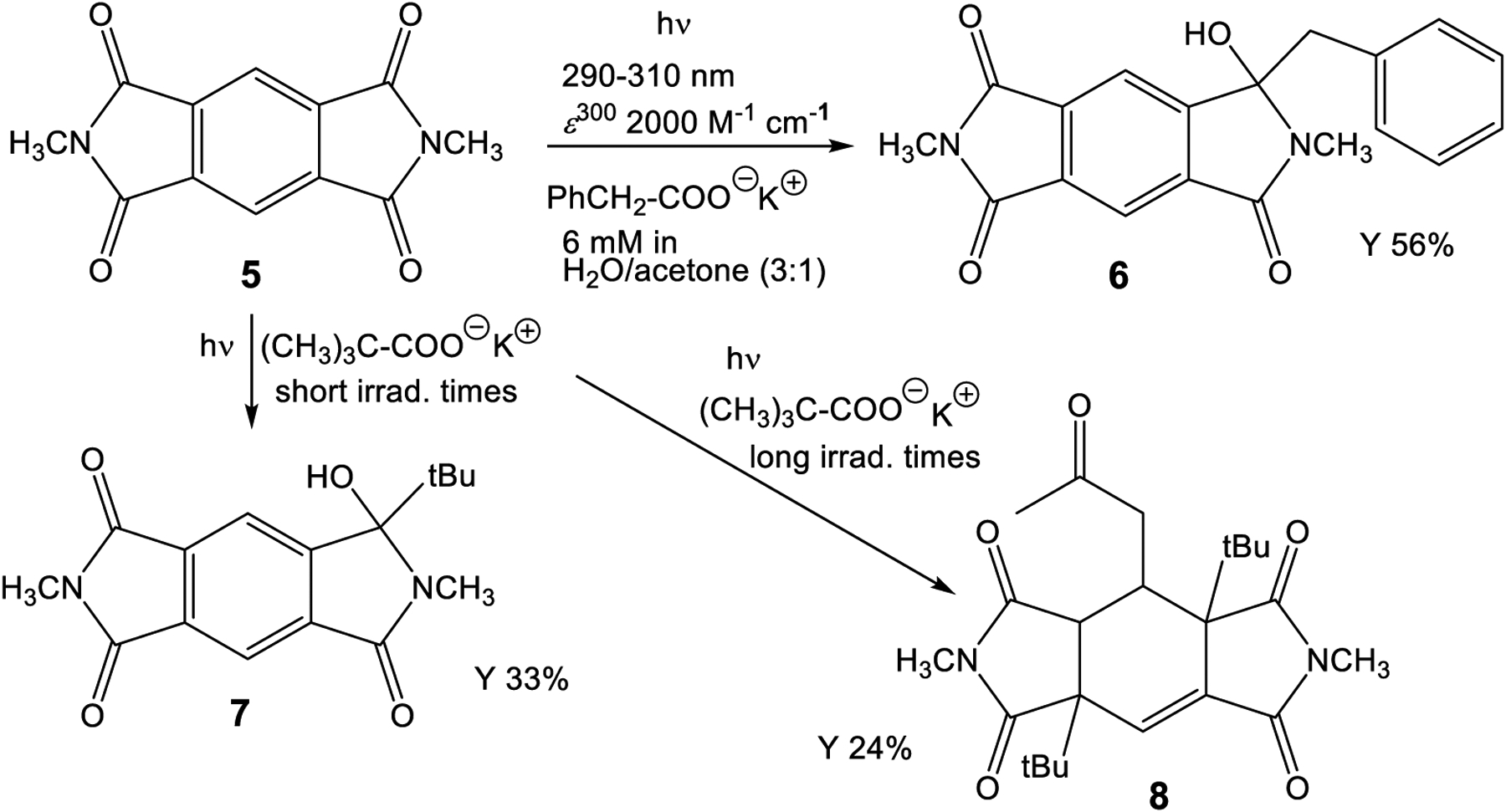
Photoreaction of *N,N*′-dimethyl pyromellitic imide **5** (in twofold excess) with potassium alkyl carboxylates leading to the monoalkylation products **6**, **7** and the threefold addition product **8**.

The intermolecular photochemical reactivity of the model substrate *N,N*′-dimethyl pyromellitic imide **5** with alkyl carboxylates under standard conditions resulted in mono- and bis-alkylation products. A specific example is the benzylation of **5** in the photoreaction with the carboxylate of phenylacetic acid (Scheme 2). In this case, predominantly the mono-adduct **6** was isolated even if twofold excess of phenylacetate is applied. Apparently, the efficiency of the second electron transfer/decarboxylation process is strongly reduced in comparison to the first one. Similarly, the use of potassium pivalate gave rise to the formation of the mono-adduct **7** after short irradiation times (less than 6 h). After extended irradiation times, the structurally unusual and complex addition product **8** was isolated for the potassium pivalate reaction with *N,N*′-dimethyl pyromellitic imide **5**: double addition of the *tert*-butyl radical on the aromatic carbons with subsequent trapping of a solvent molecule of acetone. The molecular structures from X-ray crystallography (Figure 2) confirmed the NMR data which showed only one proton in the sp^2^-region (see Supplemental Materials). When the separated and purified mono-addition product **7** was again irradiated under the standard conditions, **8** and starting material **5** were formed, suggesting a dissociation step. Similarly, irradiation of **6** led to disappearance of this starting material, but only **5** and traces of an **8** analogue with benzyl instead of *tert*-butyl were identified in the NMR.

### 3.2. Intramolecular Reactivity

**Scheme 3.**
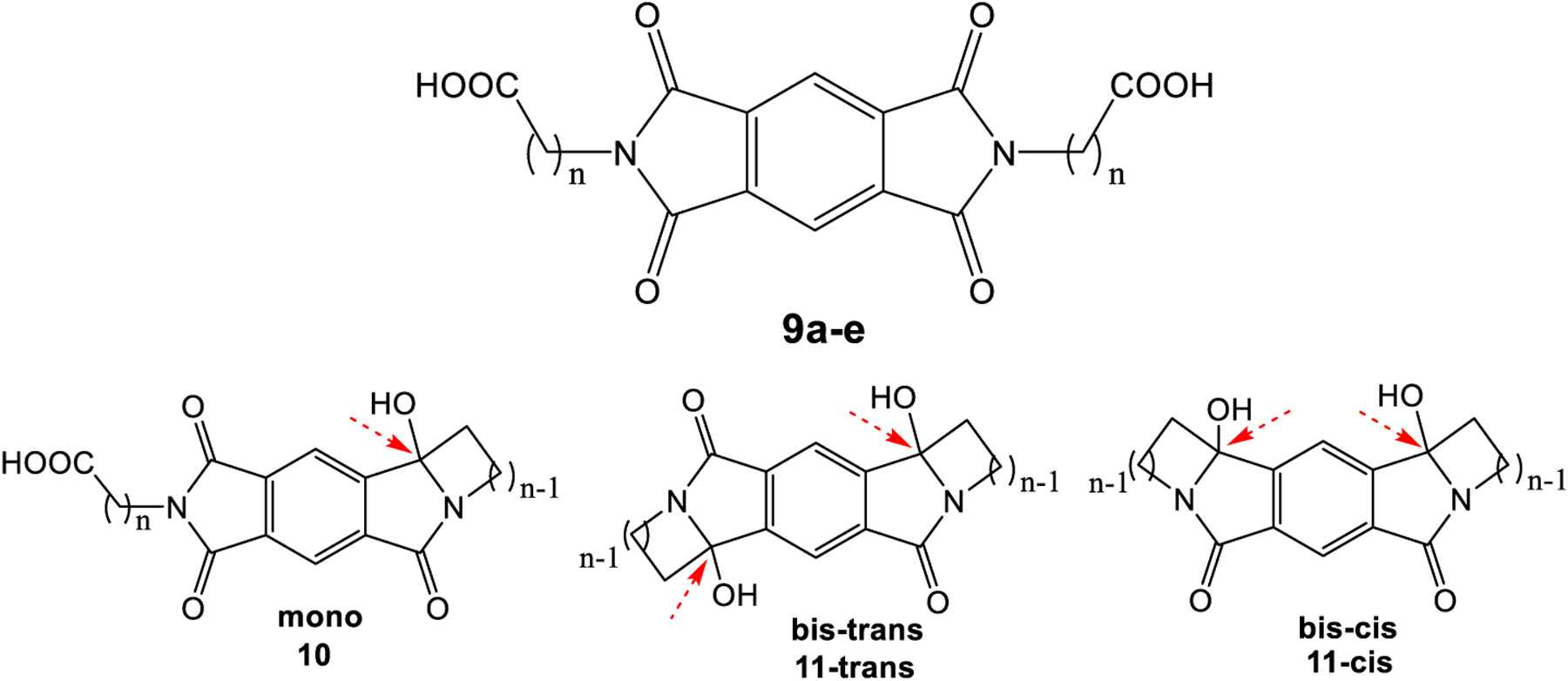
Intra-pyromellitimide substrates (**9a–e**) and possible products. The red arrows indicate the newly formed quaternary carbons with characteristic ~90 ppm ^13^C NMR peaks.

**Scheme 4.**
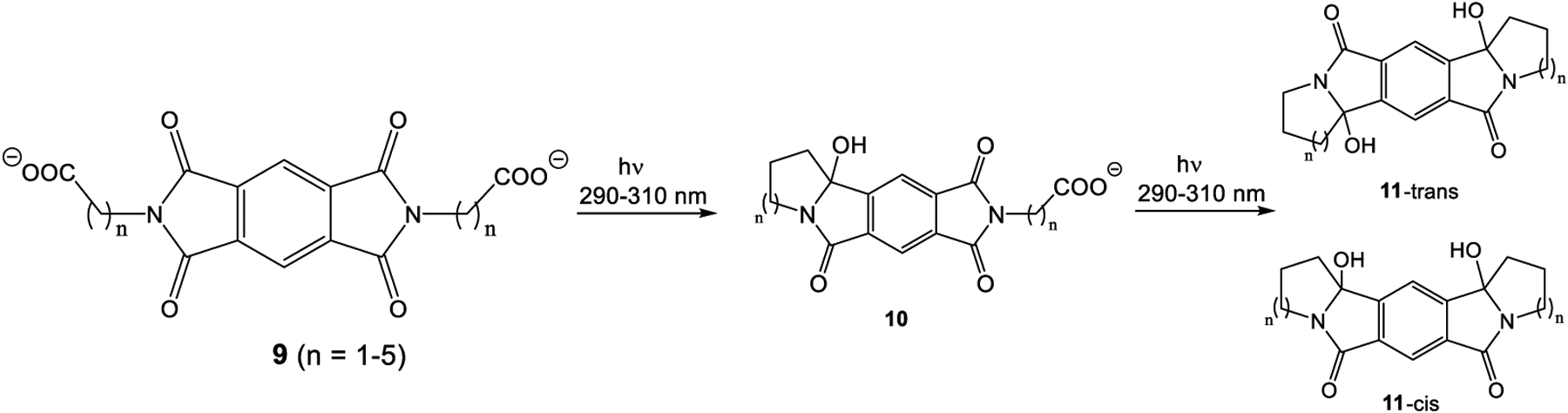
Photoreaction of **9c–e**. Stepwise cyclization.

**Scheme 5.**
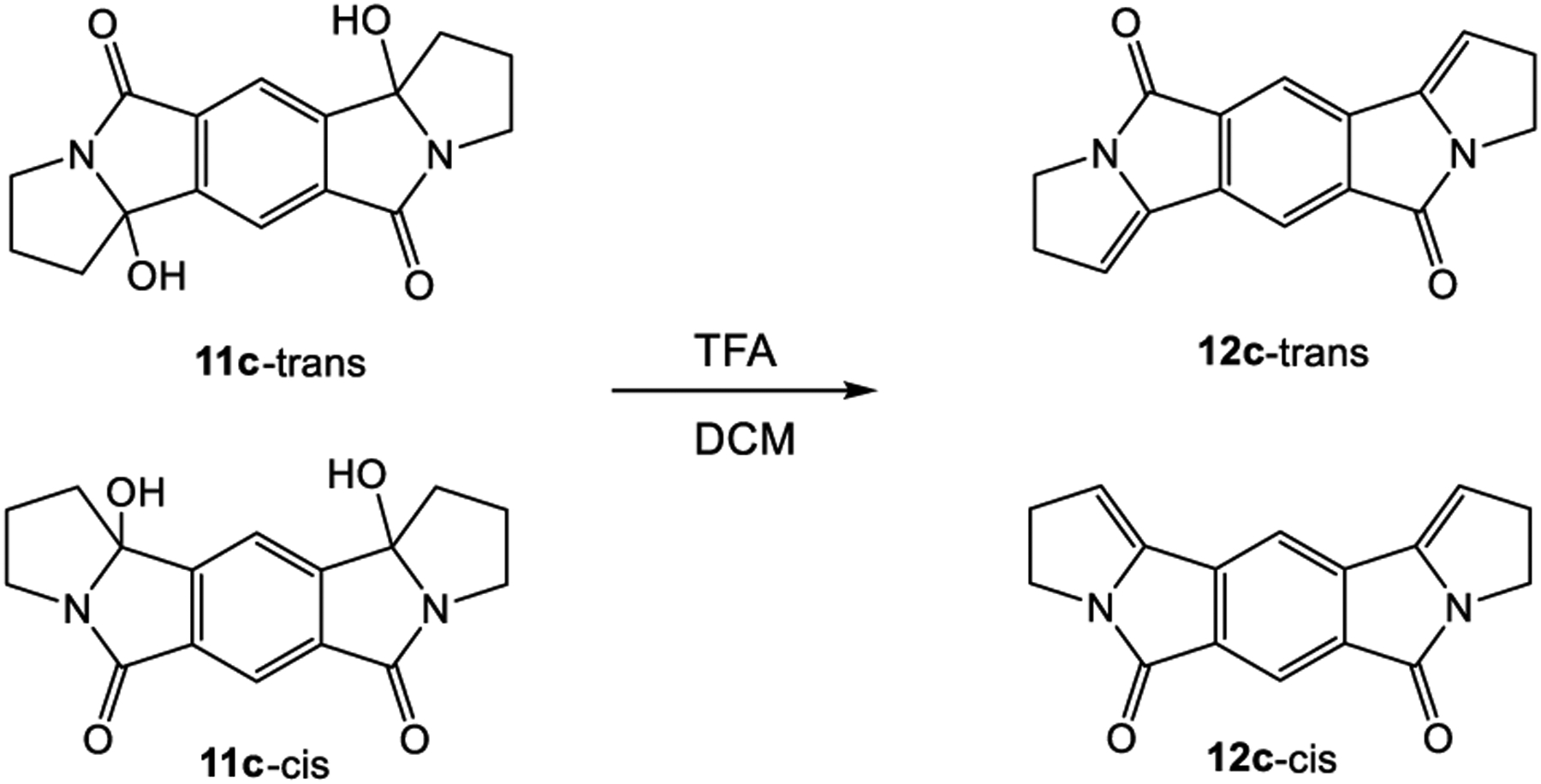
Identification of the regioisomers of **11c** (photoproduct of **9c**) by elimination with trifluoroacetic acid.

In order to test for intramolecular reactivity, we synthesized the five pyromellitimide derivatives **9a**-**e** from pyromellitic acid anhydride and glycine (**9a**, n = 1), *β*-amino propionic acid (**9b**, n = 2), *γ*-amino butyric acid (**9c**, n = 3), *δ*-amino pentanoic acid (**9d**, n = 4), *ε*-amino hexanoic acid (**9e**, n = 5). For the substrates **9c**–**9e** (n = 3, 4, 5) the reaction mixtures after photolysis in an acetone/water solvent mixture and extraction showed the characteristic ~90 ppm peak in the ^13^C NMR, which typically corresponds to the newly formed quaternary carbon centers (see arrows in Scheme 3, and Supplemental Materials). This indicated the formation of cyclization products analogous to the phthalimide photochemistry. Basic and acidic extraction yielded the mono (**10**) and bis-cyclization (**11**) products, respectively (Scheme 4). Cyclization on both sides of the substrate diimide however gives rise to a large number of regio- (**11**-trans and **11**-cis) and stereoisomers. Attempts to separate them were unsuccessful; via NMR methods we were able to distinguish at least three isomers. The product mixture of **10c** was treated with TFA to destroy the stereocenter at the former carbonyl carbon. Chromatographic separation of the two products allowed unambiguous NMR identification of the bis-cis elimination product **12c**-cis (Scheme 5).

**Figure 3.**
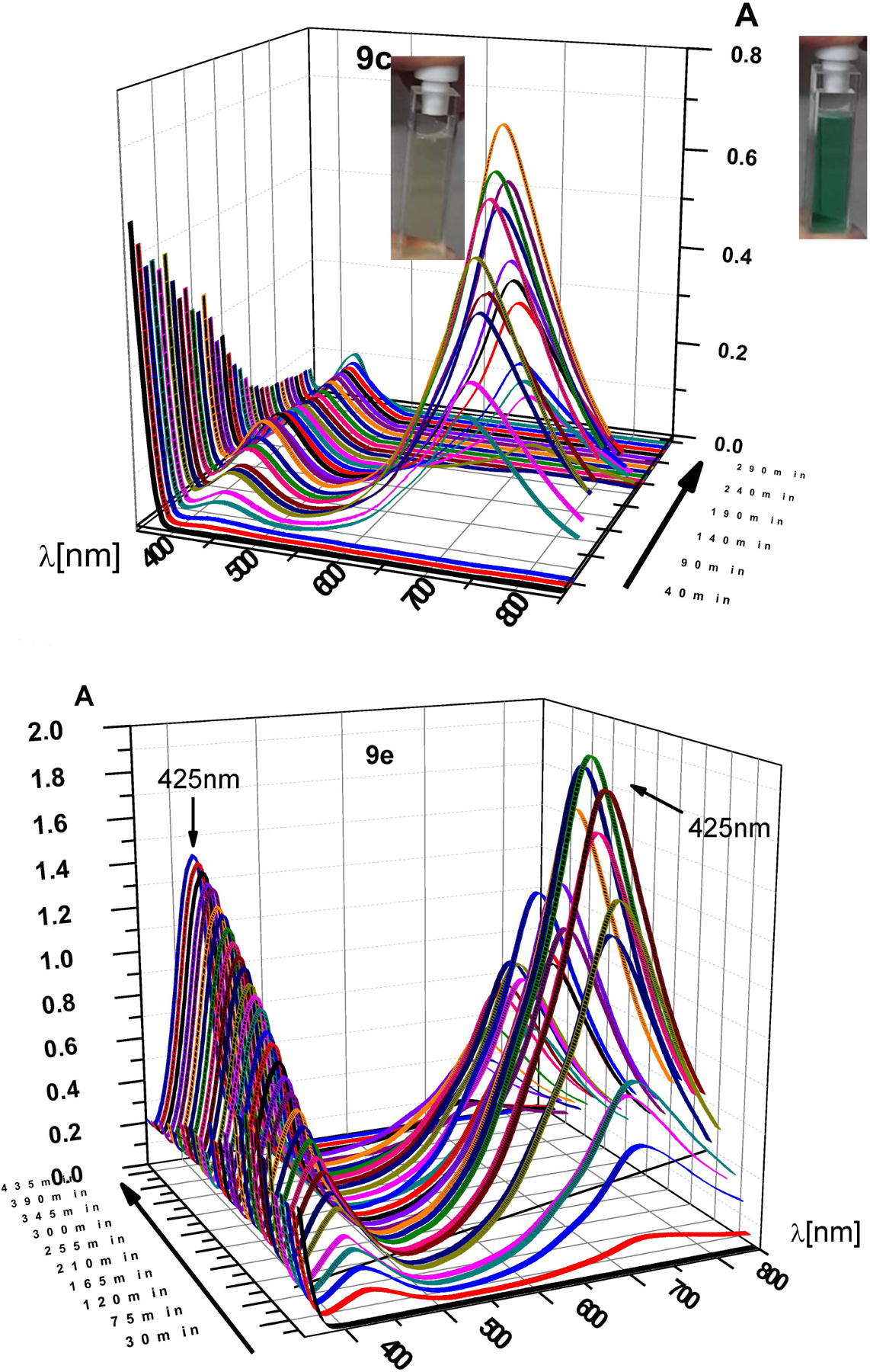

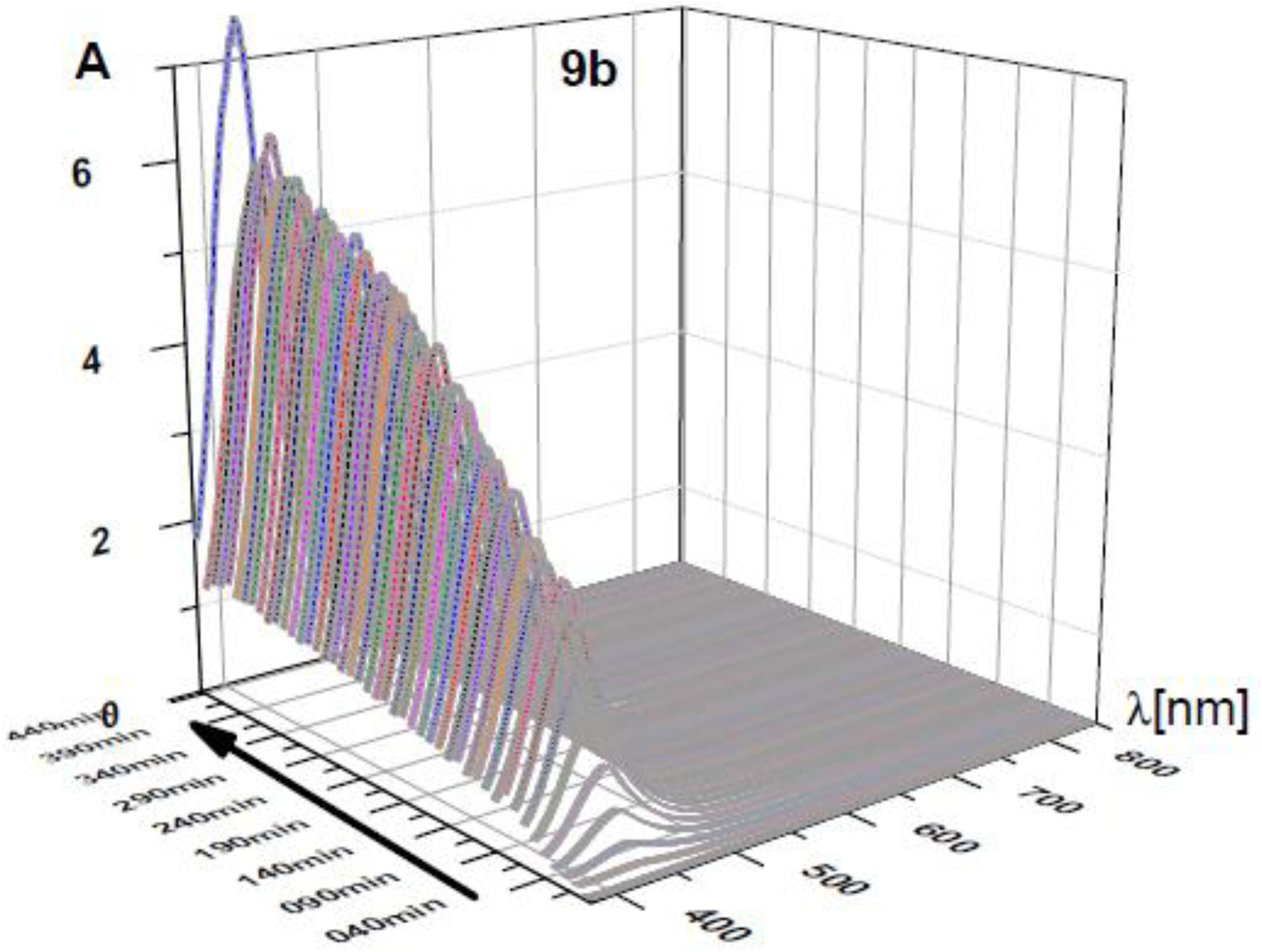
UV-vis time course of photoreactions of **9e**, **9c**, and **9b** under constant irradiation at *λ* = 300 nm. Vertical axis: absorbance. Note that the absorbance scale is different for all three graphs (max values: for n = 2 max. *A* = 8, for n = 3 max. *A* = 0.8, for n = 5 max. *A* = 2.0). **9e** and **9c** show very similar time-dependent behavior and are shown in side and front view. **9b** does not form a long-lived 715 nm absorbing species (pyromellitic diimide radical anion). The solutions of **9e** after short and long irradiation time (green and faint yellow, respectively) are also shown.

For n = 1, 2 there seemed to be fewer products, similar to the phthalimide cases. For n = 1 the simple decarboxylation product (*N,N*′-dimethyl pyromellitic diimide) was isolated in small yields. In addition, a complex product precipitated during the acidic extraction workup step. Particularly for n = 2, only a complex compound precipitated during the workup after irradiation. To gain more information about the differing reaction pathways we monitored the UV-vis absorption over the course of the reaction (Figure 3). As inset in Figure 3 for compound **9e** the colored solution of the radical anion is shown.

**Figure 4.**
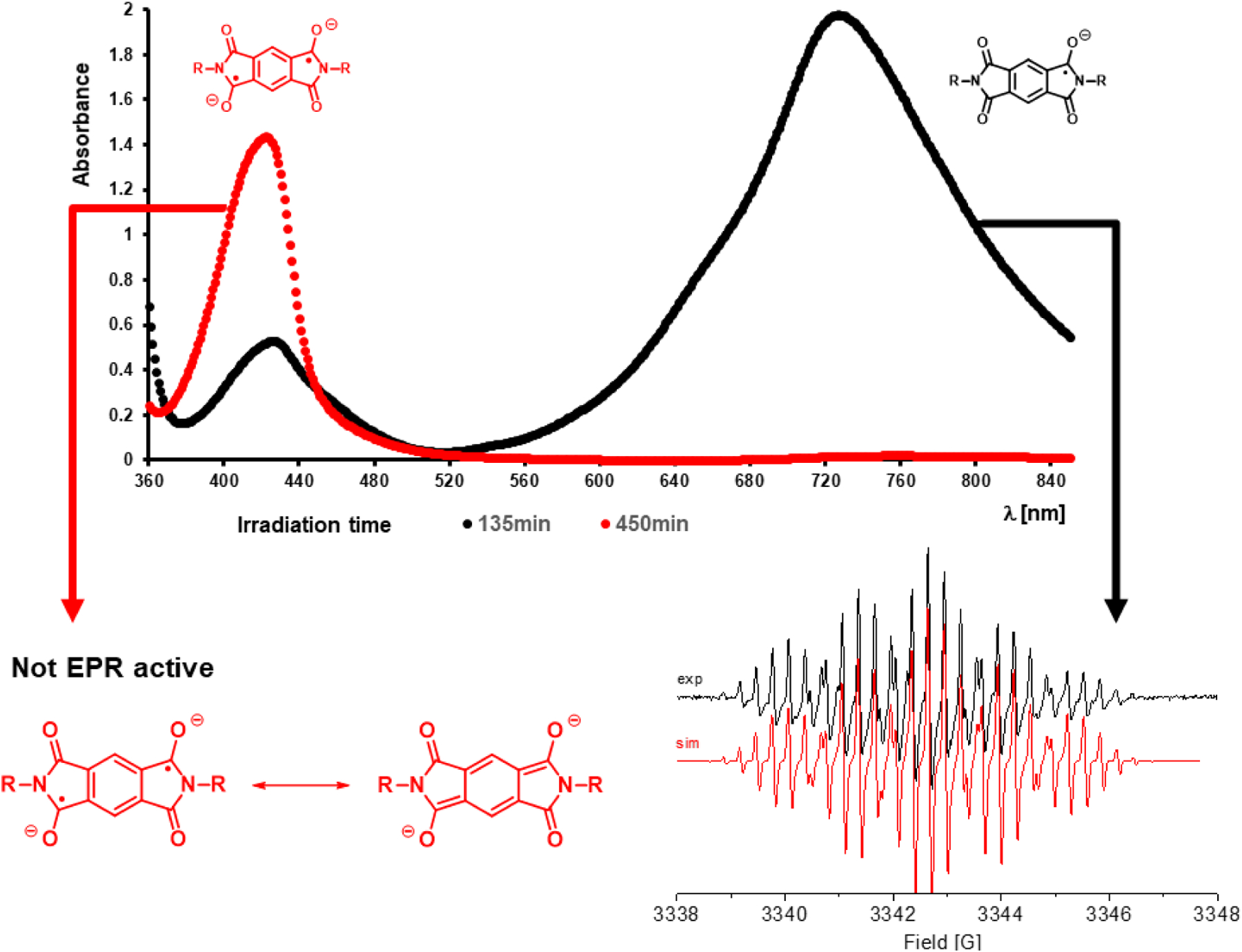
UV-vis spectrum of irradiation solution of **9d** (n = 4) after 135 min (black curve, radical anion) and 450 min (red curve, dianion). The solution at 135 min is EPR active as expected, shown X-Band EPR spectrum of (conc. = 12.4 mM) in H_2_O at 298 K with simulation (in red). The spectrum was simulated using *g* = 2.015 and HFS coupling with 2 × N = 1.29 G, 2 × H(arom.) = 0.33 G, and 2 × H = 0.31 G, 2 × H = 0.30 G, 2 × H = 0.29 G, 2 × H = 0.28 G, line width of 0.05 G and Lorentzian/Gaussian = 1.0. 450 min irradiation solution is not EPR active.

**Figure 5.**
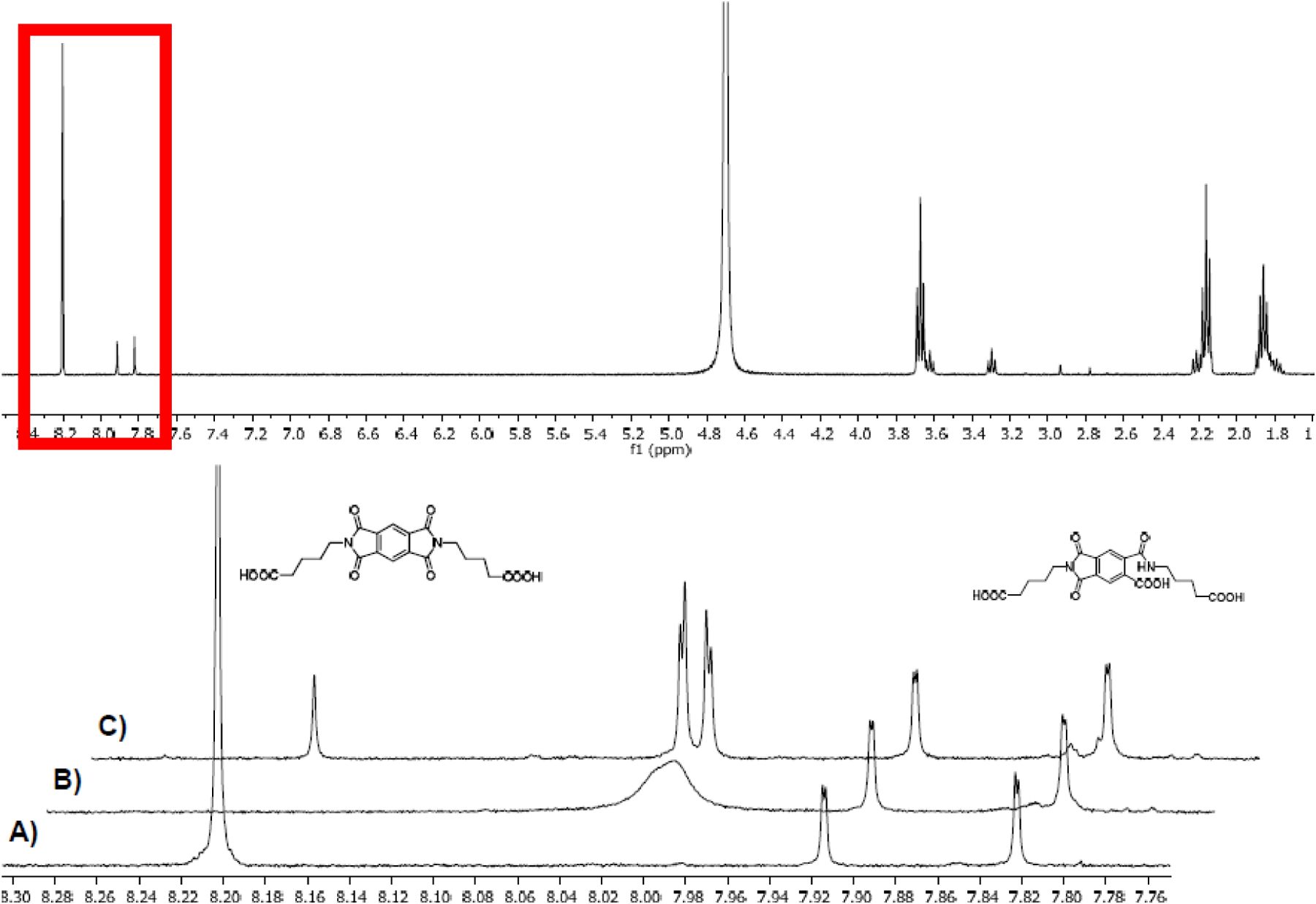
NMR spectra of **9d** (approx. 7 mM, in D_2_O/K_2_CO_3_), top spectrum: full ^1^H NMR, stacked: zoomed into the 7.75–8.35 ppm region. Pyromellitimide protons at 8.20 ppm. Peaks at 7.91 and 7.82 ppm are caused by the hydrolysis product of **9d** (structure shown) under the basic conditions and conveniently serve as internal standard. (**A**) Before irradiation (**B**) after 10 min irradiation (**C**) sample from (**B**) after quenching with air.

Two absorptions at longer wavelengths than pyromellitimide appeared at 425 and 725 nm, respectively. The strong absorption at 725 nm (the solution became dark green after about 90 min of irradiation), which occurred for compounds with n = 3, 4, 5 can be attributed to the radical anion of pyromellitimide [33]. This species has a lifetime of hours under those conditions and has been confirmed by EPR (Figure 4) and NMR (line broadening due to paramagnetic behavior, Figure 5). The simulation of the EPR spectrum (Figure 4, in red, coupling constants with imido N and aromatic/aliphatic H given in the legend) matches perfectly the experimental spectrum, proving that the radical anion is symmetric with respect to the *N*-side chains and the carboxylic acids are still intact. The simulation of the decarboxylation/protonation product from **9d** that would bear two *N*-C_4_H_9_ side-chains clearly differs from the experimental EPR spectrum (see also the Supplementary Material).

This radical anion can then undergo radical combination with the decarboxylated chain to yield the cyclization products. In addition, an absorption at 425 nm was observed, which is assigned to the double-reduced quinoid-like structure. The absorption maximum of the dianion is reported at 552 nm in DMF [33], and the shift to 425 nm can be explained by protonation in aqueous solution. This structure presumably leads to unexpected coupling products on the aromatic ring (vide infra) and is the only absorption visible for compounds with n = 1, 2 (but also exists for n = 3, 4, 5). The 425 nm species consequently does not exhibit an EPR signal. The long lifetime of the radical anion is surprising, particularly considering the fate of the primary alkyl radical that must have formed concurrently after electron transfer and decarboxylation. The hydrophobic cores of the diimides associate in aqueous solution and thus exhibit increased stability due to through-space coupling. Using the reported extinction coefficient (*ε*((PI^*•*−^)) = 41,700 M^−1^ cm^−1^ [33]), it can be estimated that about 1% of the total pyromellitimides exist as radical anion at the peak during the irradiation (between 70 and 160 min, depending on n). For the shorter spacers (n = 1, 2), the decreased flexibility of the carboxylate chain leads to increased repulsion and less aggregation. For n = 1, a second electron transfer yields the simple decarboxylation product, which starts to precipitate, and the irradiation solution becomes less transparent. Particularly for n = 2, the radical anion gets reduced quickly to the dianion, which is partly protonated in aqueous solution and thus does not exhibit its typical UV-vis absorption (*λ*_max_(PI^**2−**^) = 552 nm [33], Figure 3, n = 2, vertical axis scale is at an absorbance of about 8, corrected for dilution of the irradiation mixture). Radical combination now probably occurs on the aromatic ring in those quinoid structures and leads to products like the ones isolated for the intermolecular cases (vide supra).

## 4. Discussion

### 4.1. Intermolecular Reactions

The intermolecular photoreactions were conducted in solvent mixtures with a high acetone content (75%, compared to only less than 10% for the intramolecular reactions) due to the insolubility of **5** in water. Under these conditions, solvent excitation and triplet energy transfer to **5** is dominant and generates triplet excited **5**. The monoaddition products **6** and **7** are the primary photoproducts that correspond to the phthalimide photochemistry. They can easily be identified by their characteristic ^13^C NMR signals at about 90 ppm for C_q_(OH). Compound **7** is unstable and can regenerate the photochemically active radical precursor. Subsequent excitation leads to the unusual addition to the benzene ring with concomitant loss of aromaticity in **8**, a reaction never observed in phthalimide photochemistry. Typical phthalimide photoproducts **2** and **4** (Scheme 1) do not exhibit photochemical reactivity (at 300 nm) but the monoaddition product **7** of **PI** reacts further, presumably by intramolecular hydrogen abstraction to dissociate the original adduct.

**Scheme 6.**
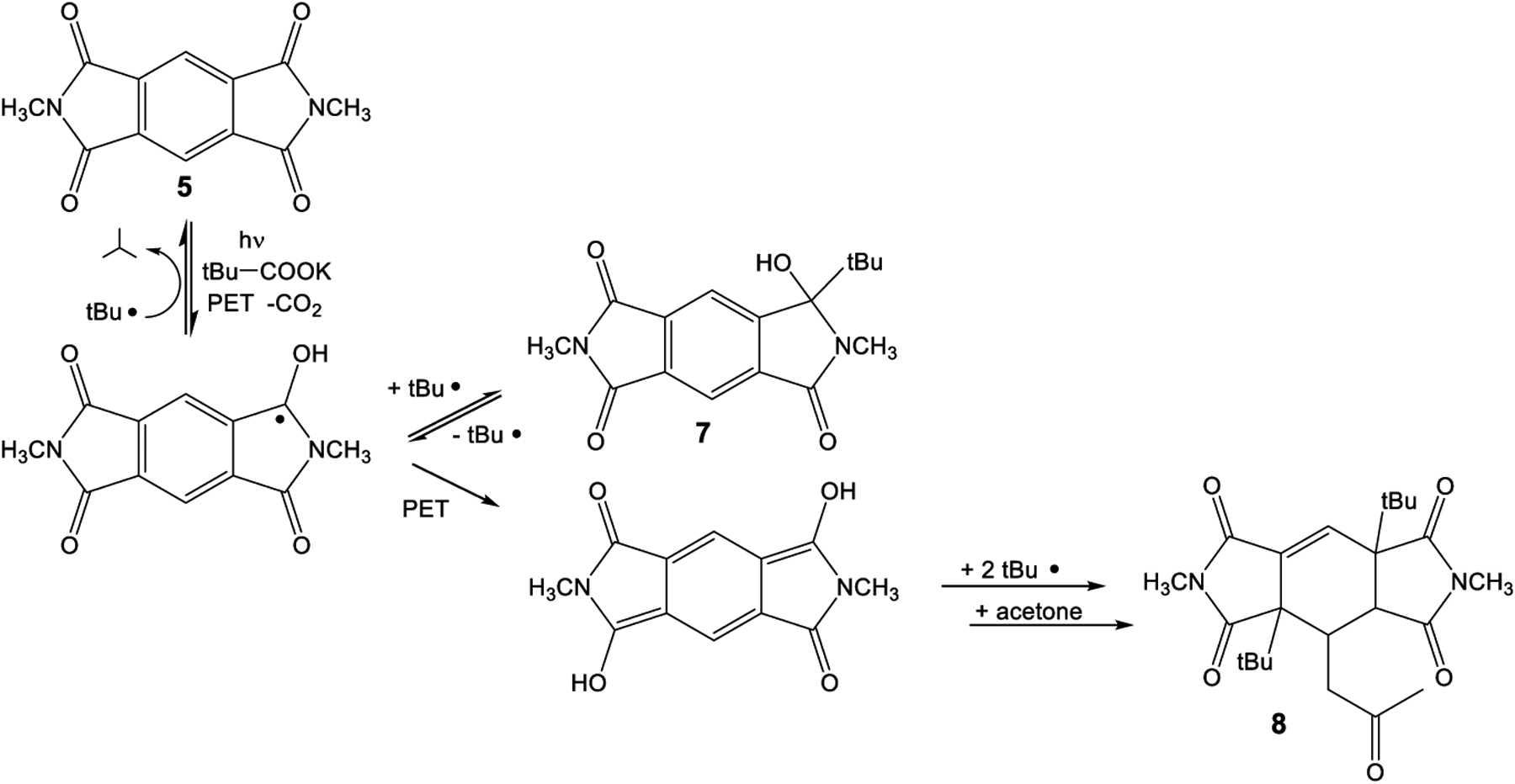
Photoreaction of **5** with pivalic acid, formation of primary and secondary products.

As a possible reaction mechanism for the formation of **8**, the following scenario is postulated (Scheme 6): after long irradiation times, the persistent radical anion can get trapped by a second electron transfer to form the dianion, which likely exists in the protonated form in aqueous solution, as evidenced by the blue-shifted UV-vis absorption at 425 nm. This intermediate combines with the alkyl radical of the decarboxylated acids to form **8**, after Michael addition of an enolized solvent acetone.

### 4.2. Intramolecular Reactions

The *N*-carboxyalkylated substrates **9c**–**6e** (n = 3, 4, 5) form the expected (i.e., from phthalimide photochemistry) intramolecular cyclization products by a sequence of PET and radical combination. Low yields and the high number of regio- and stereoisomers make the reaction less appealing from a preparative standpoint. The surprising long lifetime of the pyromellitic imide radical anion (**PI**^*•*−^) is likely caused by stacking effects of the hydrophobic core in the aqueous irradiation solution [38–40]. Short side chains in **9a,b** (n = 1, 2) prevent this stabilizing effect by electrostatic repulsion already in the ground state. In the time-course experiments, no absorption peak at 725 nm ever develops for **9a,b**. In fact, the absorption of the double-reduced species (PI^2-^) at 425 nm is particularly strong for those compounds (absorbance is extrapolated to almost 8 for **9b**). For **9a**, precipitation of the double-decarboxylation product **5** is evident in the UV-vis data and makes interpretation more difficult. The lack of stabilization of **PI**^*•*−^ can lead to back-electron transfer to yield the simple decarboxylation products. Additionally, an immediate second reduction can form **PI**^**2-**^. This species can then yield addition products to the aromatic system to result in uncharacterizable polymeric structures. The polymeric structures, while uncharacterized, have potentially similar attachment points (on the pyromellitic aromatic ring) as seen in the intermolecular product **8**.

The stabilization of the **PI** radical anion (**PI**^*•*−^) does not explain the fate of the primary radical that is formed after decarboxylation. If radical combination does not occur immediately, the decay of this primary radical prevents product formation to **10** and **11**. Interestingly, no partial or full decarboxylation products (i.e., an CO_2_H/H exchange) were isolated for **9c**–**e** (n > 2) suggesting a more complex decaying mechanism.

## 5. Conclusions

**Scheme 7.**
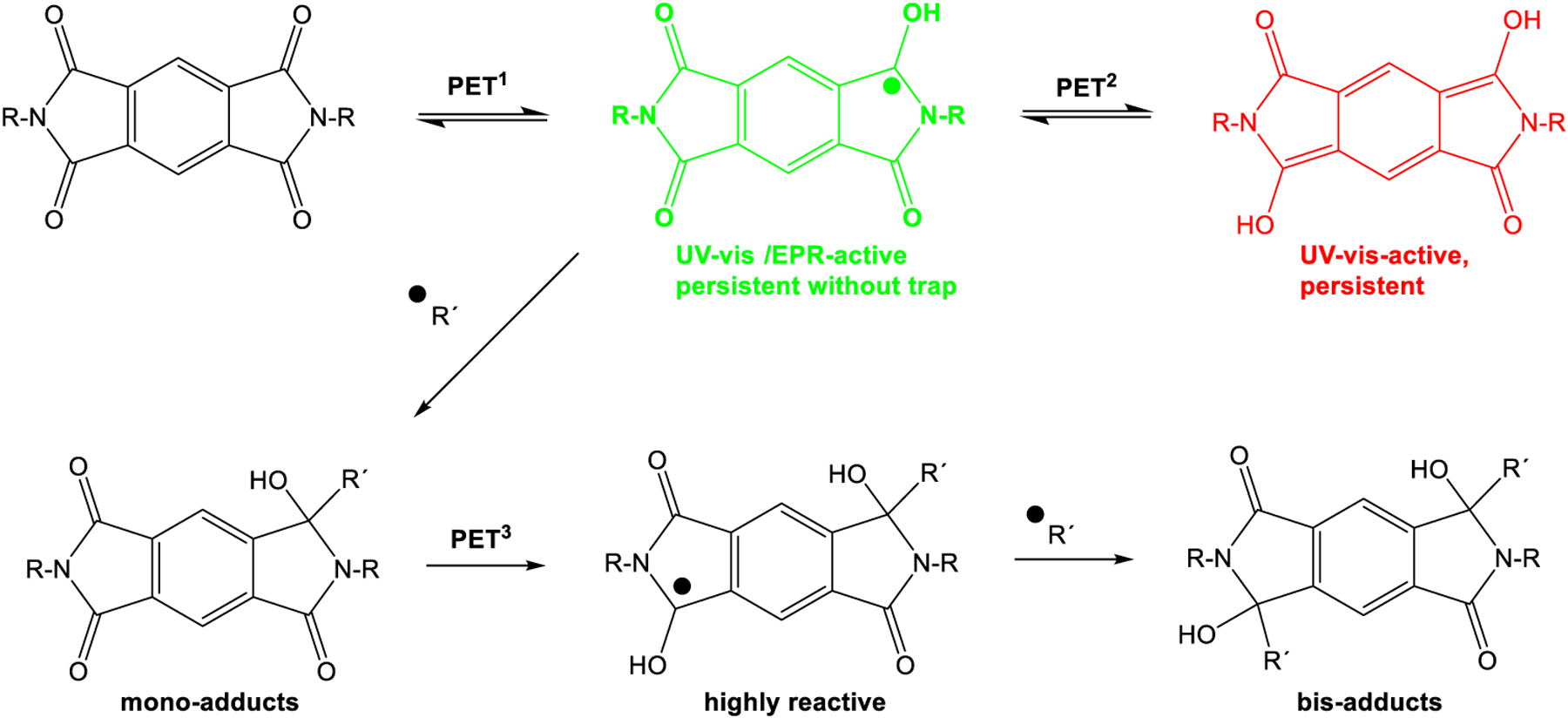
Sequential photoinduced electron transfer behavior of pyromellitic imides.

The reported stability of the pyromellitic diimide (**PI**) chromophore is the basis of numerous applications in photochemical redox systems and in polymers for mechanical and thermal protective coatings. Here, we show for the first time that **PI** exhibits surprisingly high photochemical reactivity that is analogous to the well-known photochemistry of phthalimides. The radical anion (**PI**^*•*−^) was detected by UV-vis absorption, NMR, and EPR spectroscopy in aqueous solution. In the absence of a radical source, either external or internal, the **PI**^*•*−^ is persistent and can be spectroscopically characterized or additionally reduced to the **PI**^**2−**^ (Scheme 7). This dianion appears to be even longer lived and yields products that eventually destroy the aromatic chromophore. While the product distribution is too multifaceted to make the **PI** photochemistry interesting from a synthetic point of view, it nevertheless shows that **PI** does get efficiently consumed in photoinduced electron transfer processes.

## Supplementary Material

Supplemental Materials

## Data Availability

The X-ray diffraction data for compounds **7** and **8** were transferred to the Cambridge Crystallographic Data Centre with the following deposition numbers: compound **7**: CCDC 2191095, and compound **8**: CCDC 1439632.
