# Supplementary material for: Awakening a Molecular Mummy: The Inter-and Intramolecular Photochemistry of Pyromellitic Diimides with Alkyl Carboxylates
Source: Photochem. Author manuscript; Available in PMC 2024 May 23. (PMC11115379; doi:10.3390/photochem2030046)
Supplement: Supplemental Materials [file NIHMS1944852-supplement-Supplemental_Materials.pdf]

Awakening a Molecular Mummy: The Inter-and Intramolecular Photochemistry of Pyromellitic Diimides with Alkyl Carboxylates

Wolfgang H. Kramer [1,*], Donya Razinoubakht [1], Gurjit Kaur [1], Axel Klein [2], Simon Garbe [2], Jörg Neudörfl [3], Sabrina Molitor [3], Anne Zimmer [3] and Axel G. Griesbeck [3,*]

[1] Department of Chemistry and Biochemistry, Millsaps College, 1701 North State Street, Jackson, MS 39210, USA
[2] Department of Chemistry, Faculty of Mathematics and Natural Sciences, University of Cologne, Inorganic Chemistry, Greinstr. 6, 50939 Köln, Germany
[3] Department of Chemistry, Faculty of Mathematics and Natural Sciences, University of Cologne, Organic Chemistry, Greinstr. 4, 50939 Köln, Germany
* Correspondence: kramewh@millsaps.edu (W.H.K.); griesbeck@uni-koeln.de (A.G.G.)

Supplemental Materials and Data:

Contents:

EPR Spectra and simulations: Figure S1 - X-Band EPR spectrum of **6d**

Figure S2 - EPR spectral simulations

NMR spectra of photoproducts:

Figure S3 ^{1}H- and ^{13}C-NMR spectra of **6**

Figure S4 ^{1}H- and ^{13}C-NMR spectra of **7**

Figure S5 ^{1}H- and ^{13}C-NMR spectra of **10c**

Figure S6 ^{1}H- and ^{13}C-NMR spectra of **10d**

Figure S7 ^{1}H- and ^{13}C-NMR spectra of **10e**

Figure S8 ^{13}C-NMR spectra of **11c** – 90 ppm region and selected peaks

Figure S9 ^{13}C-NMR spectra of **11d** – 90 ppm region and selected peaks

Figure S10 ^{13}C-NMR spectra of **11e** – 90 ppm region and selected peaks

NMR spectra of photochemical starting materials:

Figure S11 ^{1}H- and ^{13}C-NMR spectra of **9a**

Figure S12 ^{1}H- and ^{13}C-NMR spectra of **9b**

Figure S13 ^{1}H- and ^{13}C-NMR spectra of **9c**

Figure S14 ^{1}H- and ^{13}C-NMR spectra of **9d**

Figure S15 ^{1}H- and ^{13}C-NMR spectra of **9e**

<u>**EPR Spectra and simulations:**</u>

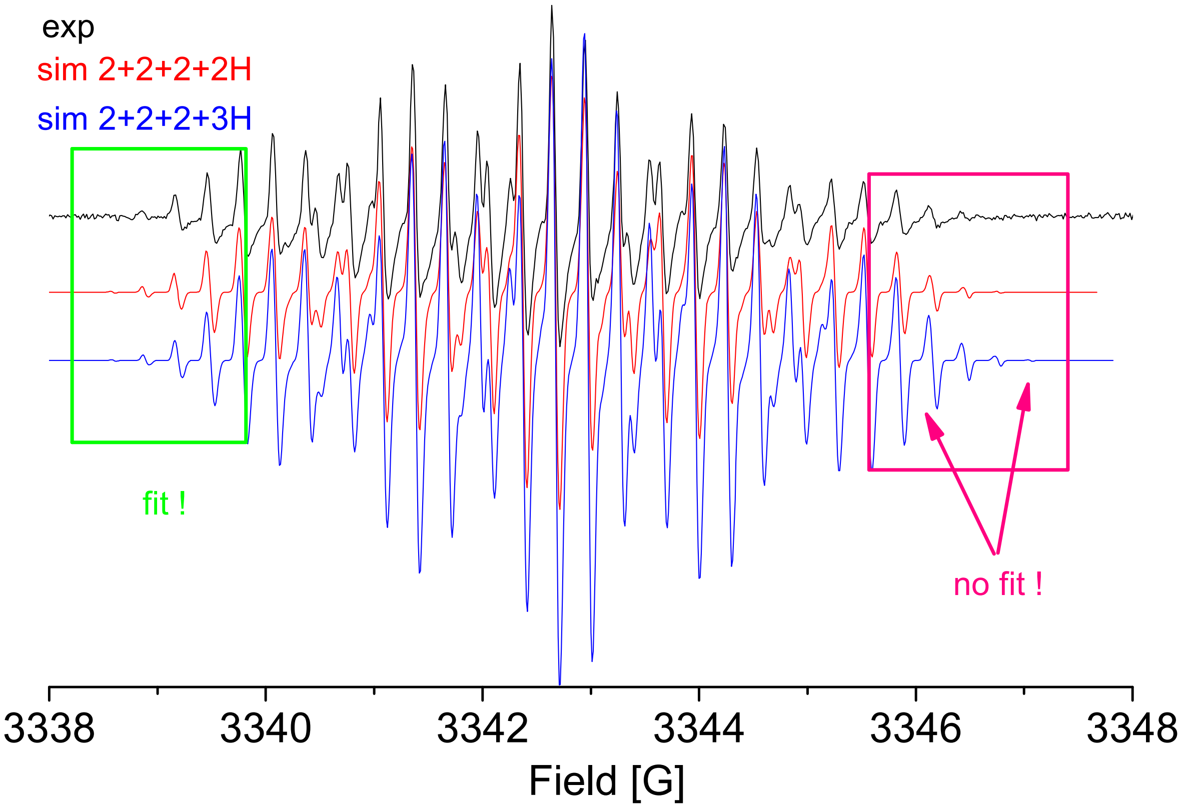

Figure S1. X-Band EPR spectrum of **6d** (n = 4)(conc. = 12.4 mM) in H_2O at 298 K with simulations containing 8H for the R group (in red) or 9H for the R group. The spectrum was simulated using *g* = 2.015 and HFS coupling with 2×N = 1.29 G, 2×H(arom.) = 0.33 G, 2×H = 0.31 G, 2×H = 0.30 G, 2×H = 0.29 G, and a) 2×H = 0.28 G (red line) or b) 3×H = 0.28 G (blue line), line width of 0.05 G and Lorentzian/Gaussian = 1.0.

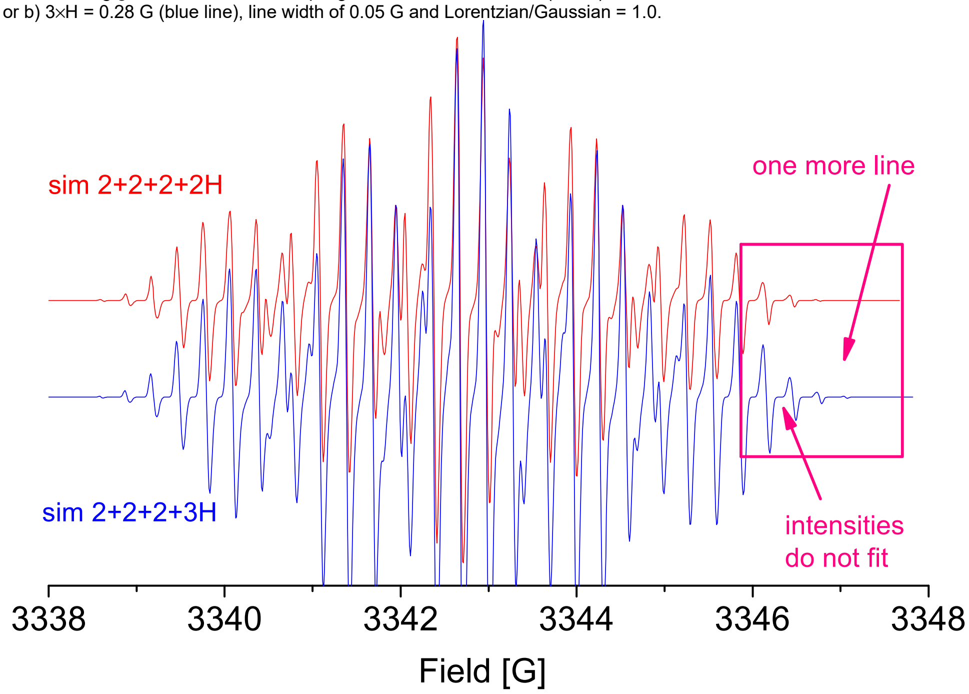

Figure S2. Two EPR spectral simulations containing 8H for the R group (in red) or 9H for the R group. The spectrum was simulated using *g* = 2.015 and HFS coupling with 2×N = 1.29 G, 2×H(arom.) = 0.33 G, 2×H = 0.31 G, 2×H = 0.30 G, 2×H = 0.29 G, and a) 2×H = 0.28 G (red line) or b) 3×H = 0.28 G (blue line), line width of 0.05 G and Lorentzian/Gaussian = 1.0.

NMR Spectra of Photoproducts:

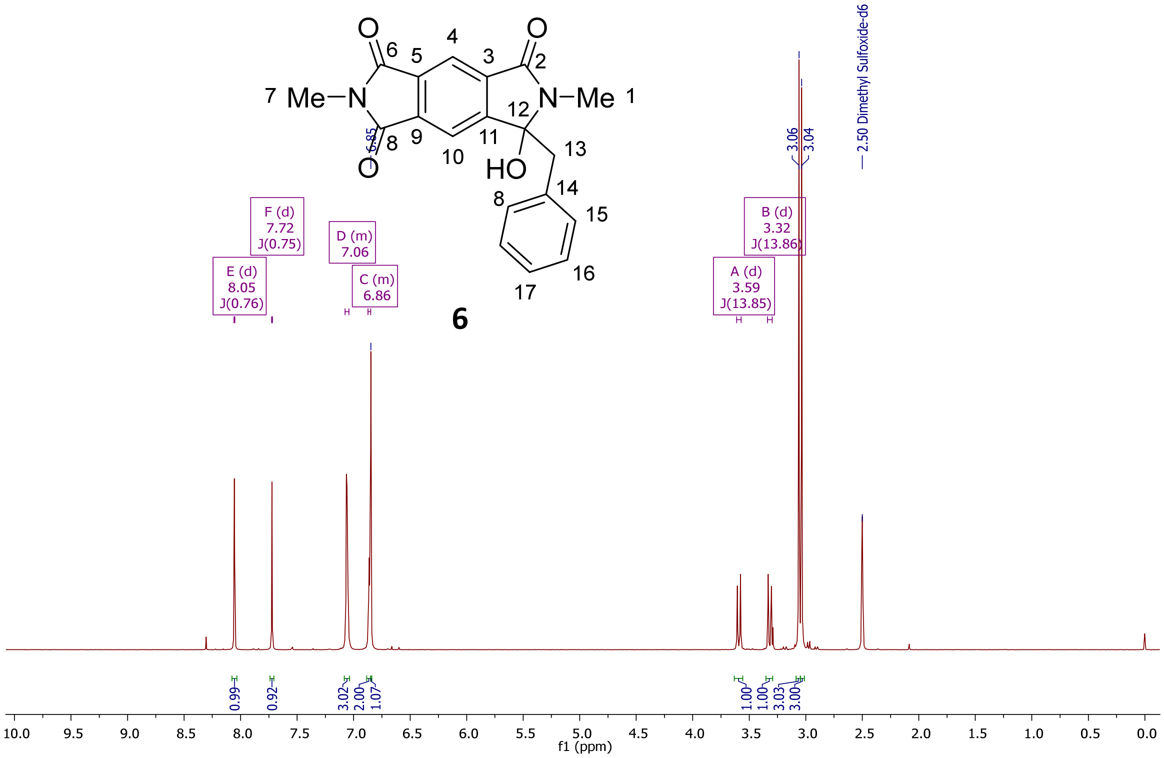

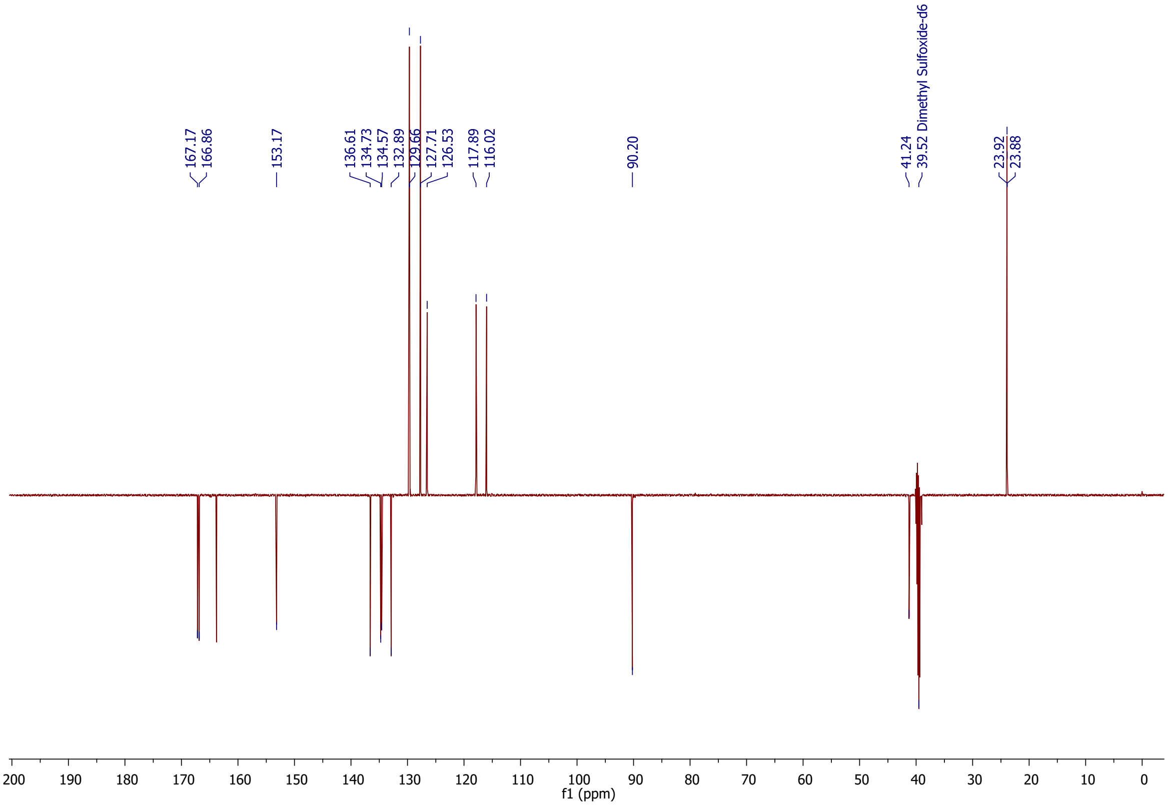

Figure S3. ^{1}H- and ^{13}C-NMR spectra of **6**

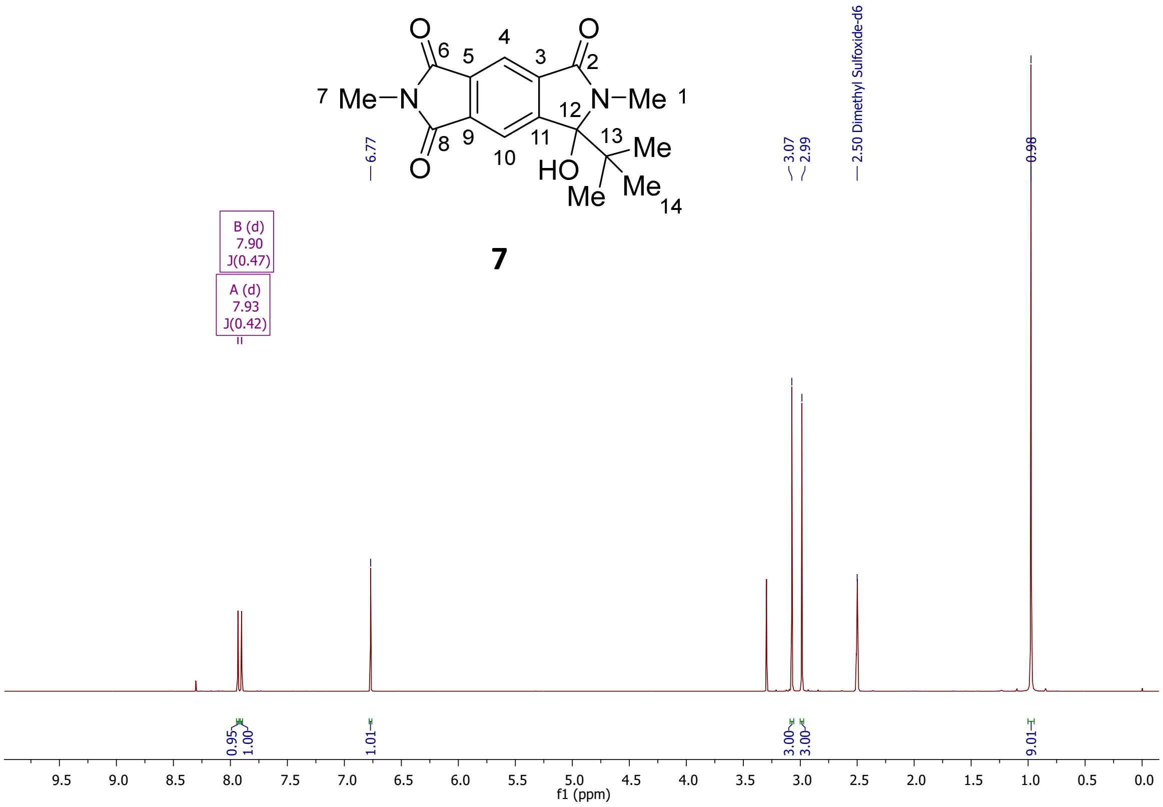

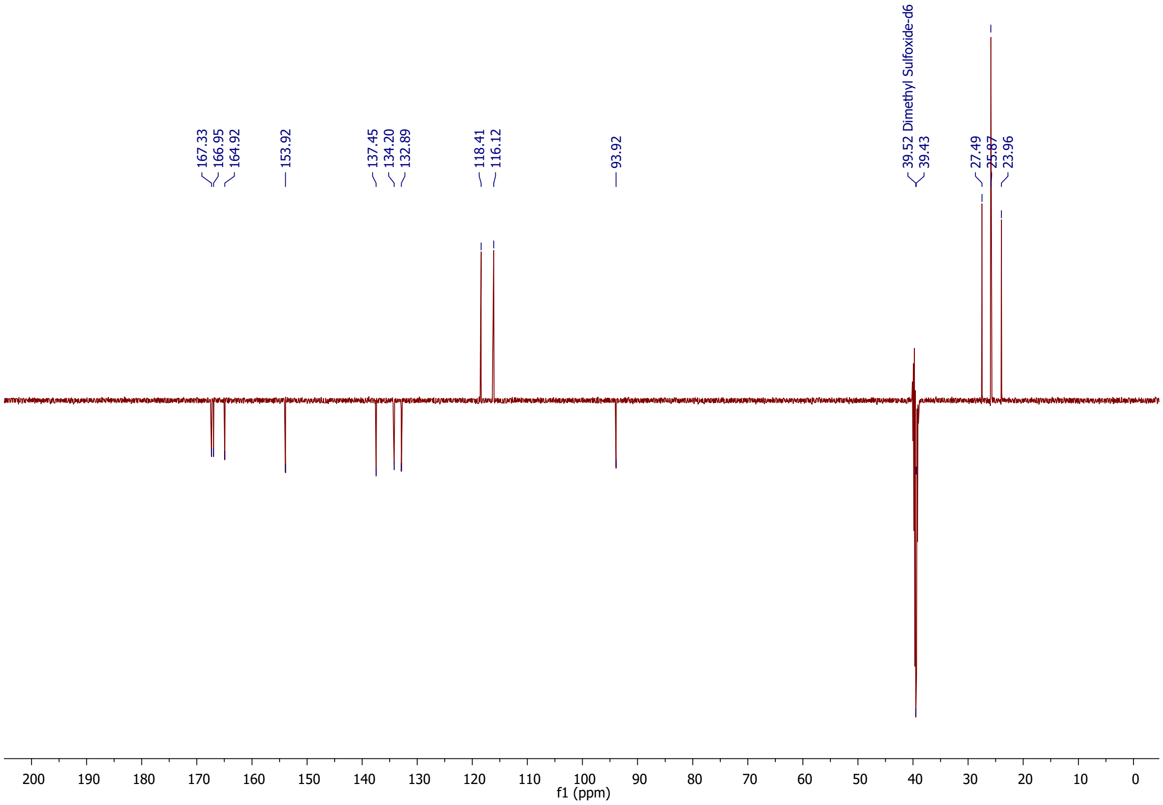

Figure S4. ^{1}H- and ^{13}C-NMR spectra of **7**

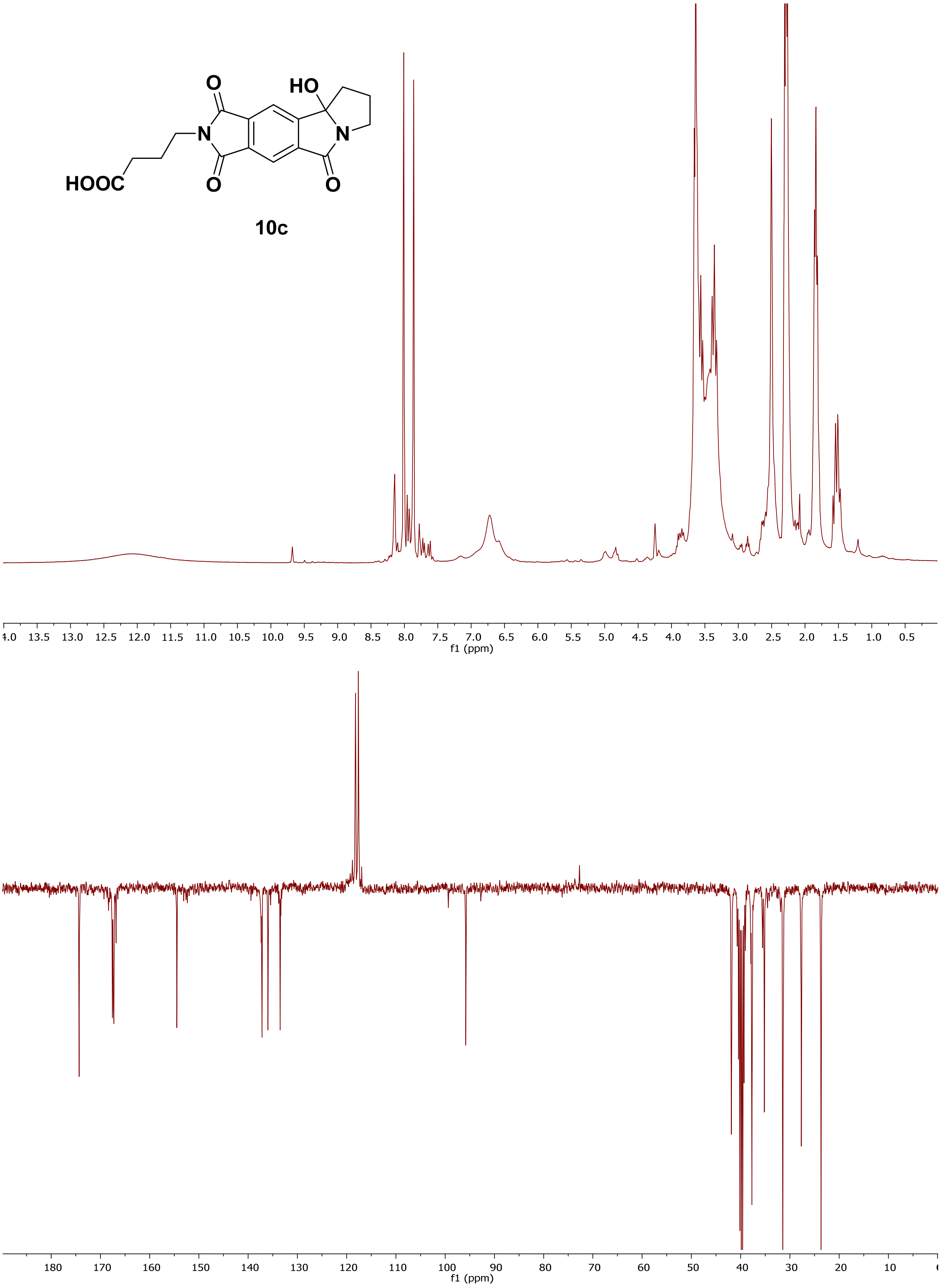

Figure S5. ^{1}H- and ^{13}C-NMR spectra of **10c**

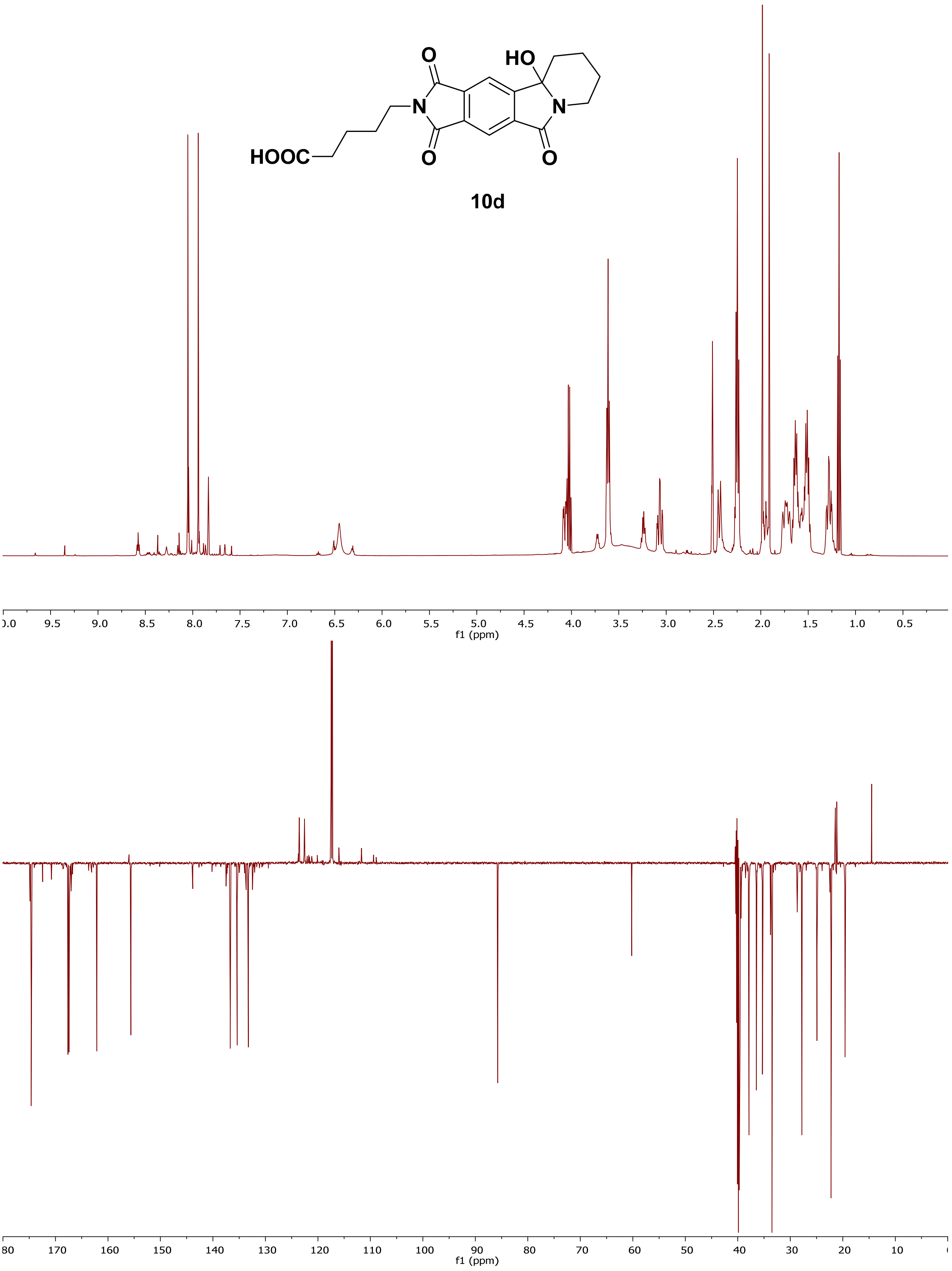

Figure S6. ^{1}H- and ^{13}C-NMR spectra of **10d**

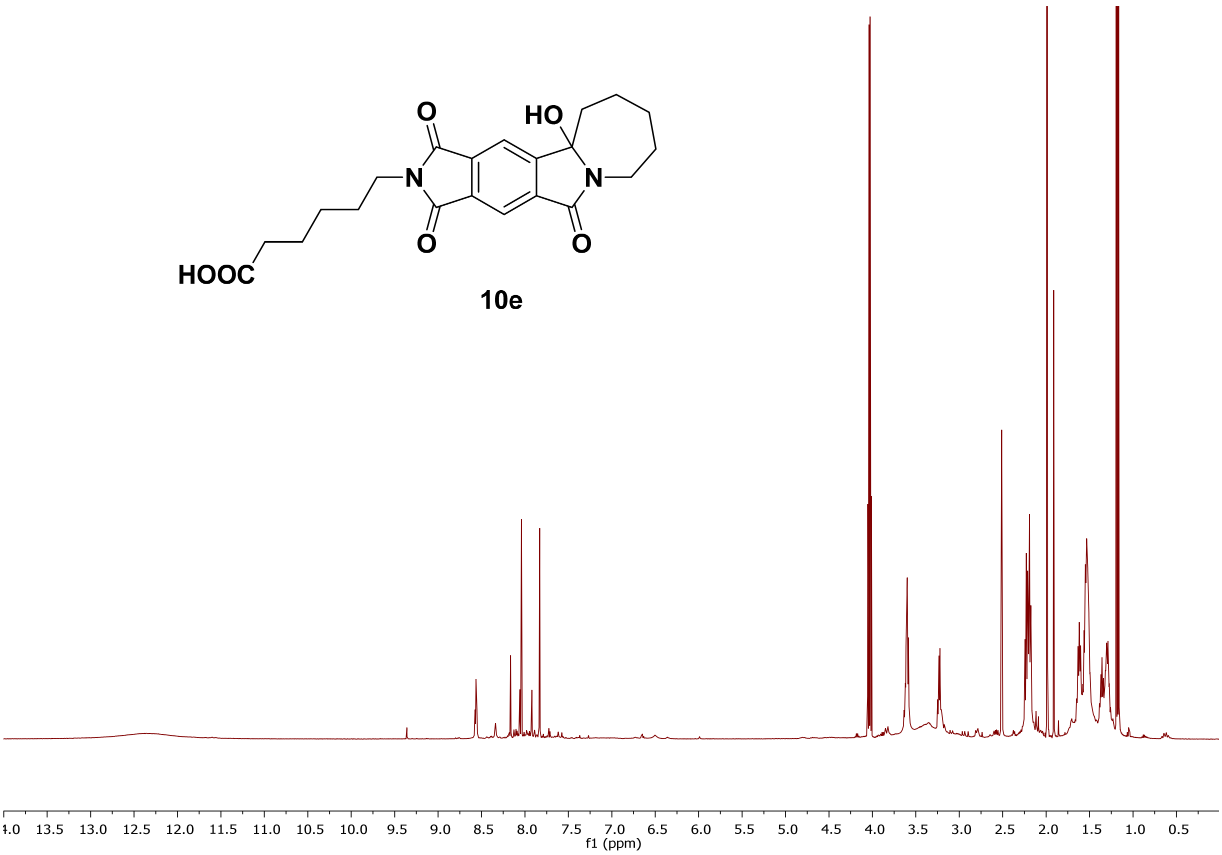

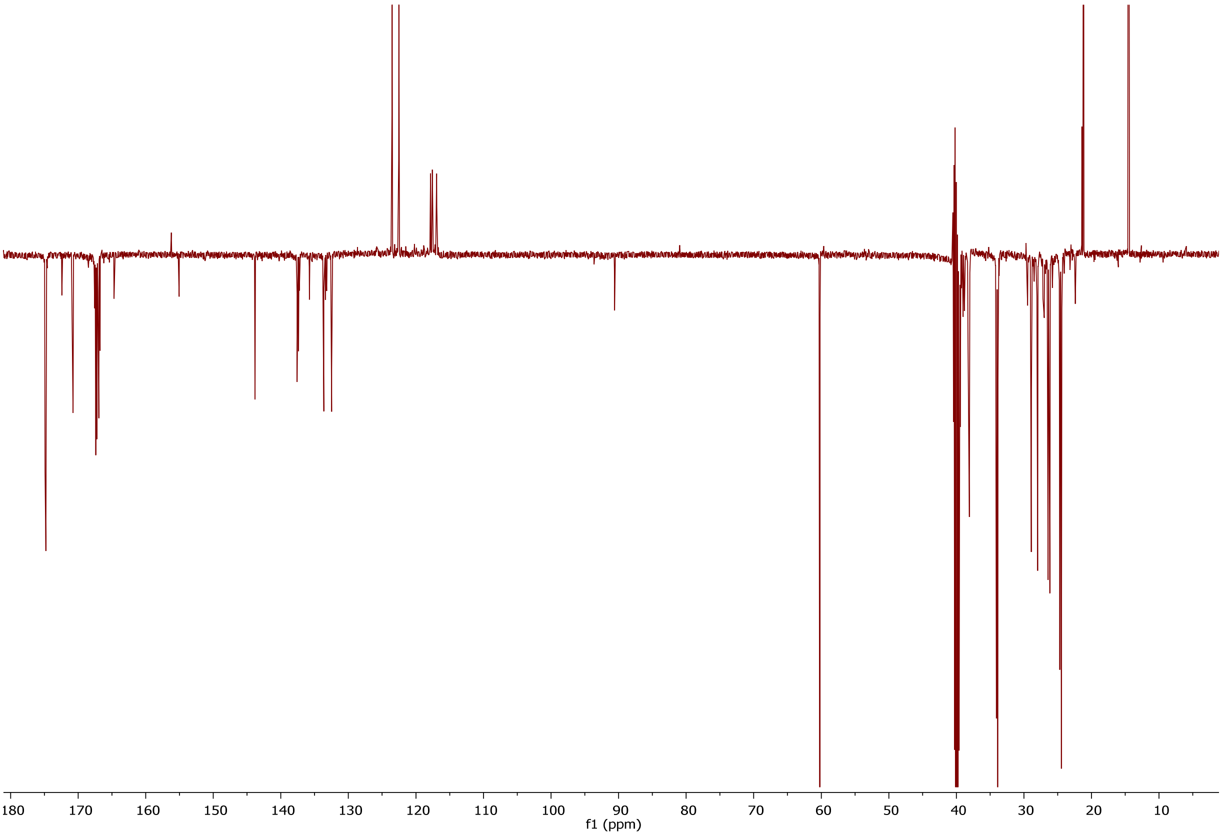

Figure S7. ^{1}H- and ^{13}C-NMR spectra of **10e**

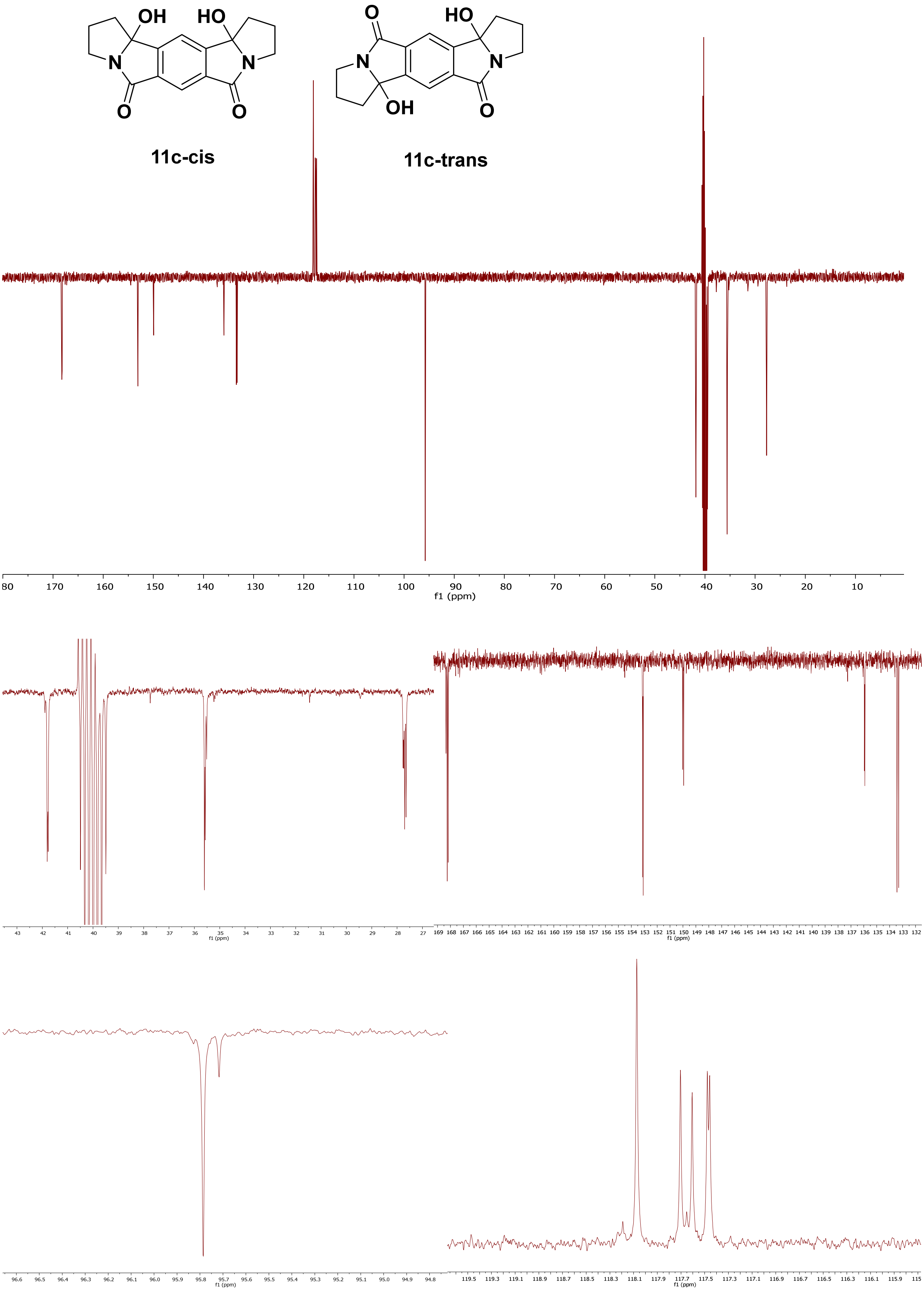

Figure S8. ^{13}C-NMR spectra of **11c** – 90 ppm region and selected peaks

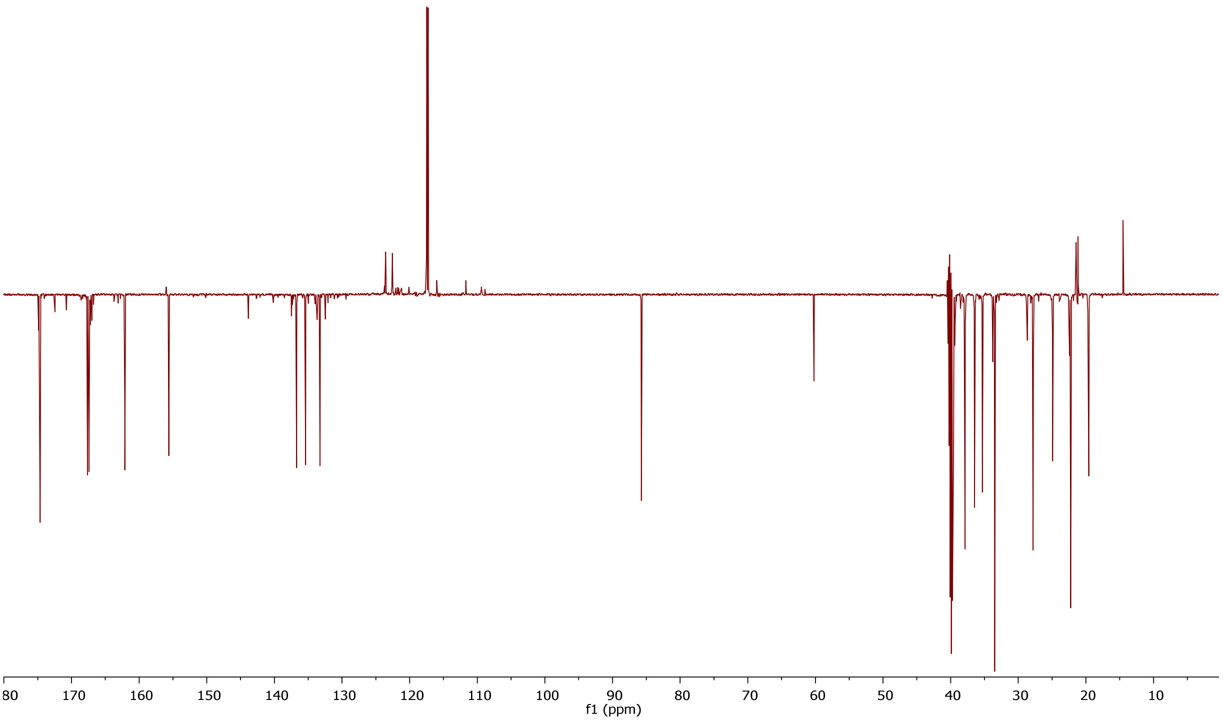

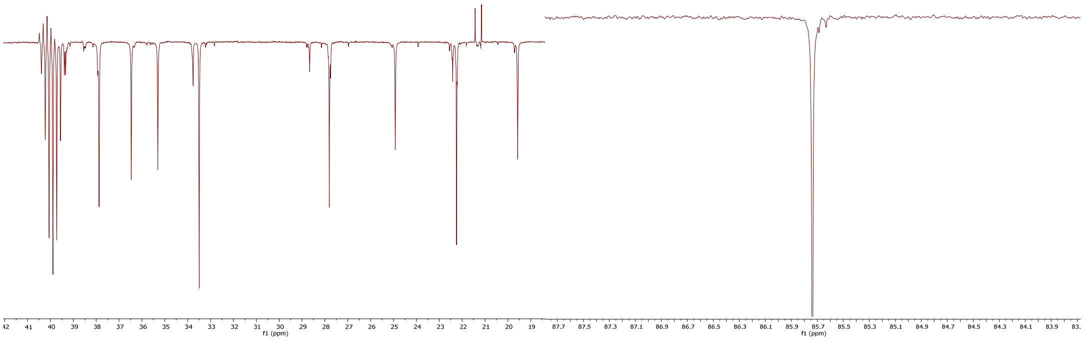

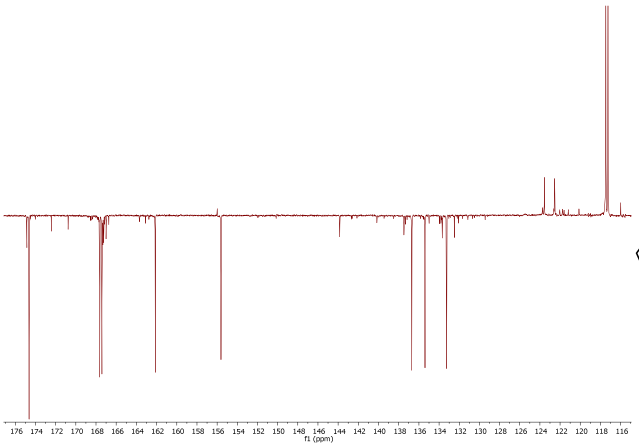

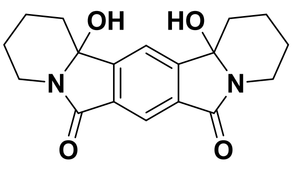

11d-cis

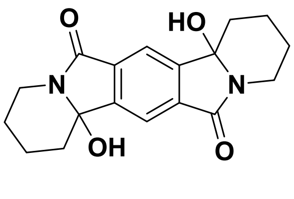

11d-trans

Figure S9. ^{13}C-NMR spectra of **11d** – 90 ppm region and selected peaks

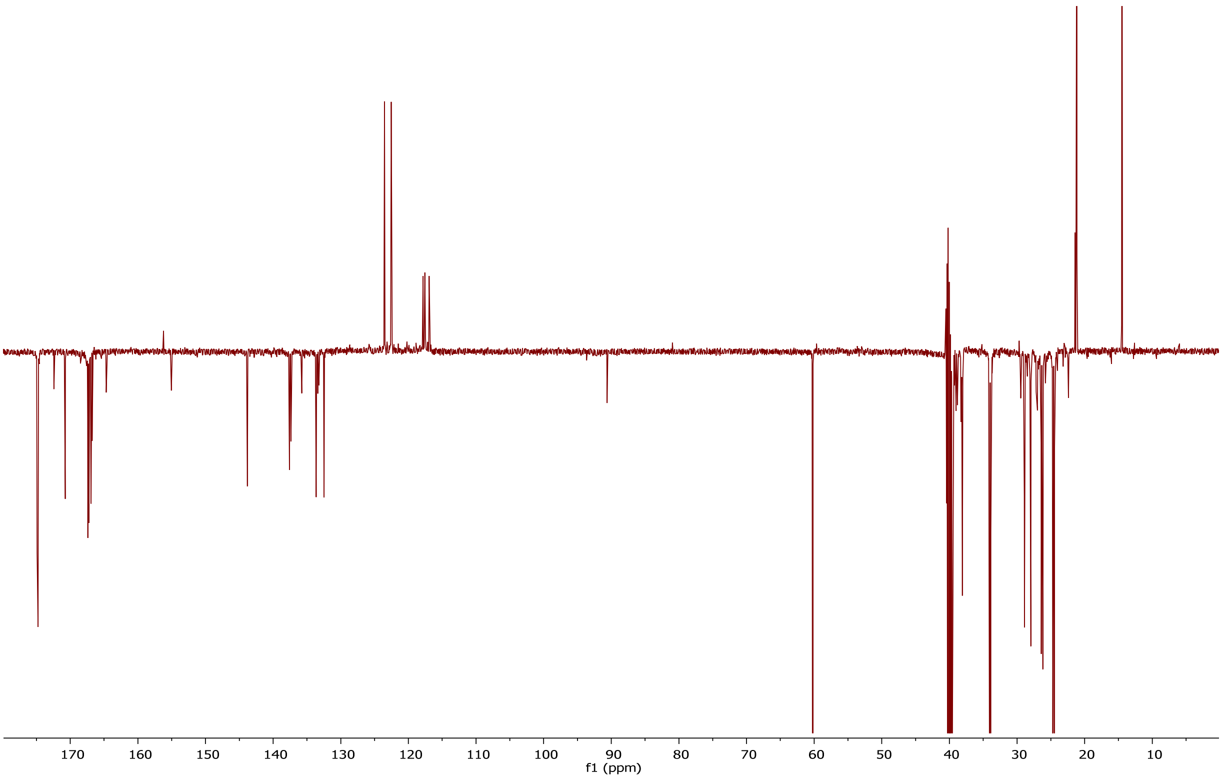

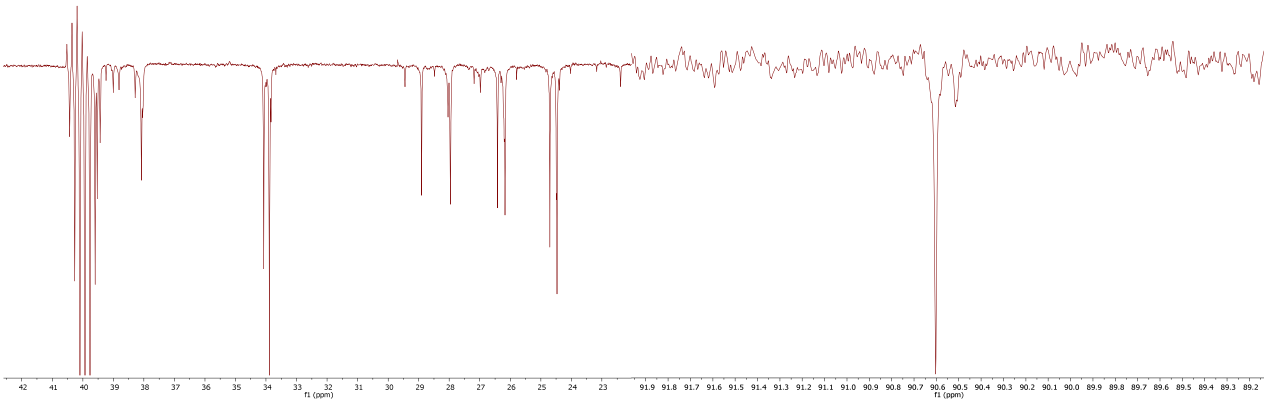

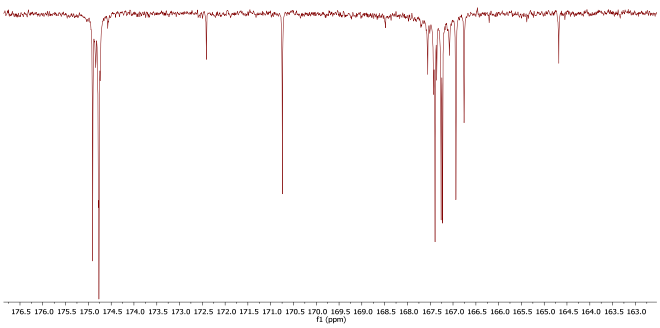

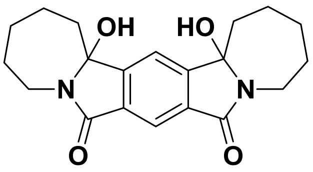

11e-cis

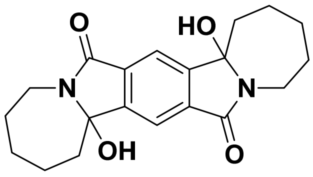

11e-trans

Figure S10. ^{13}C-NMR spectra of **11e** – 90 ppm region and selected peaks (Ethyl acetate impurity)

NMR spectra of photochemical starting materials:

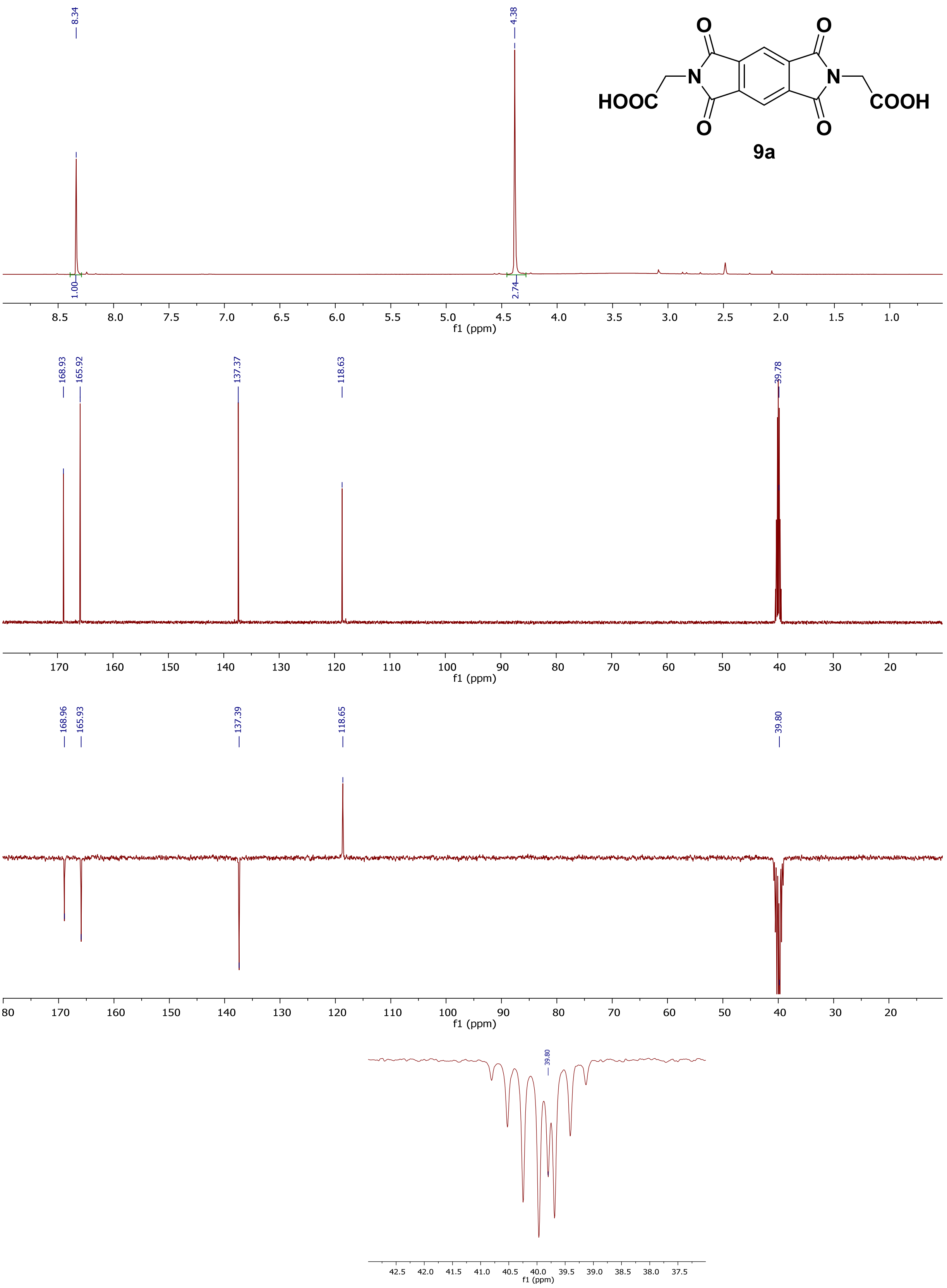

Figure S11 ^{1}H- and ^{13}C-NMR spectra of **9a** – 40 ppm region

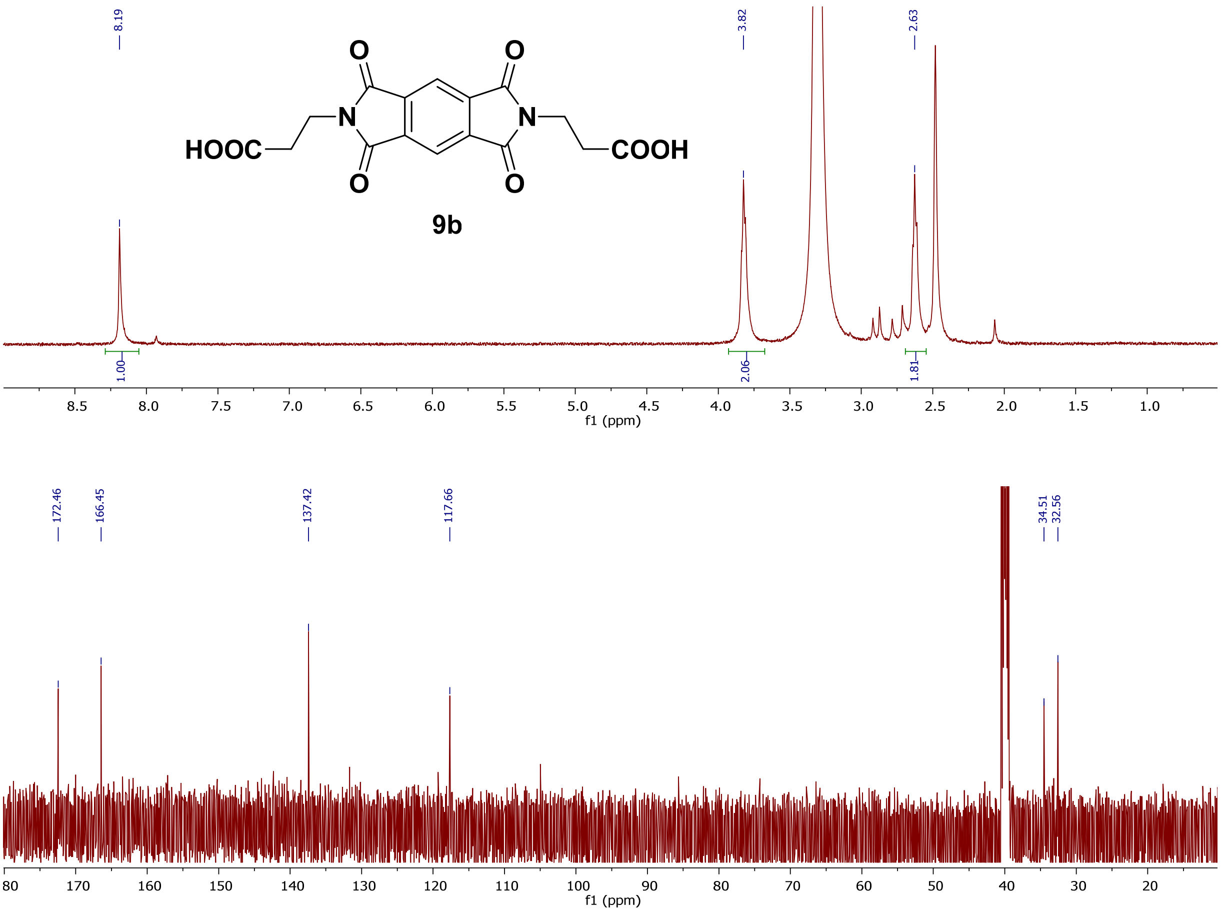

Figure S12 ^{1}H- and ^{13}C-NMR spectra of **9b**

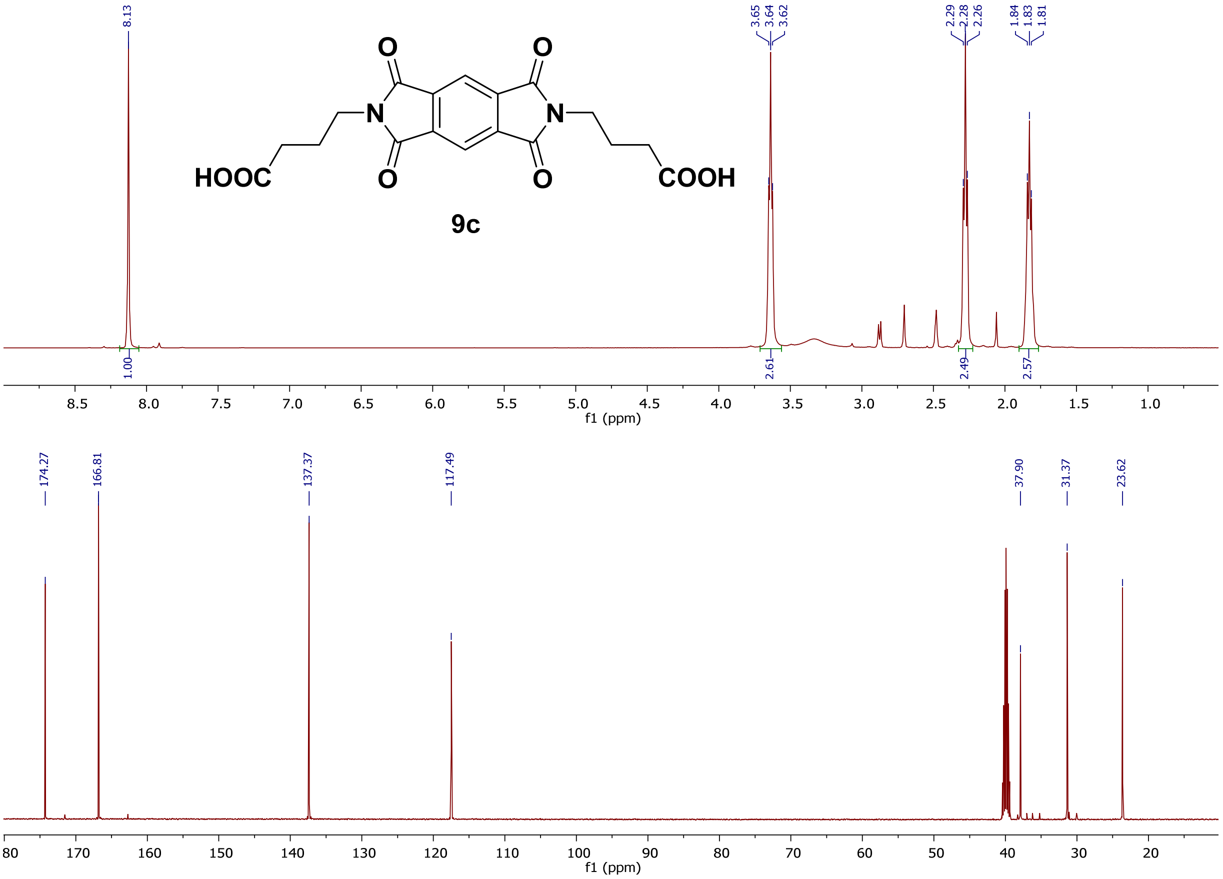

Figure S13 ^{1}H- and ^{13}C-NMR spectra of **9c**

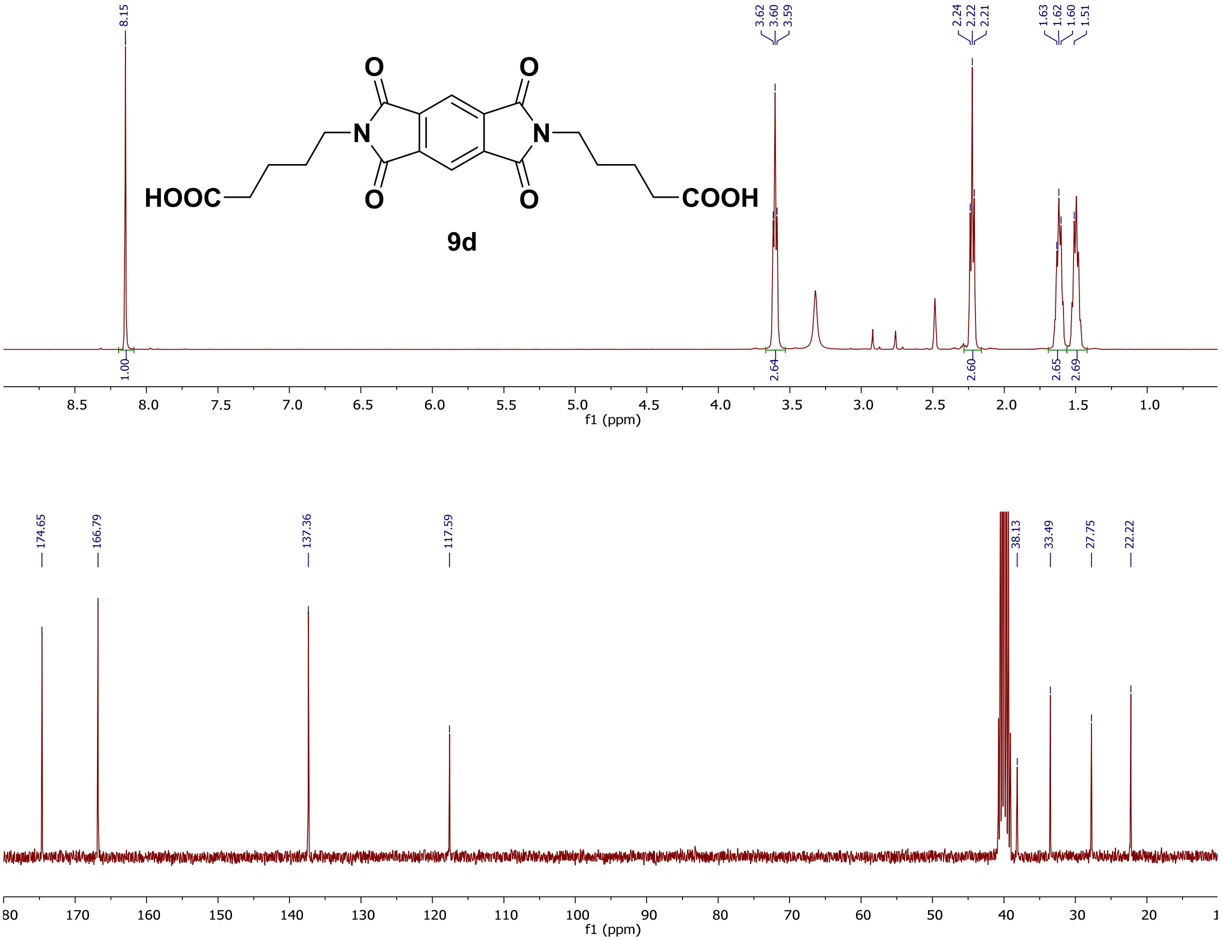

Figure S14 ^{1}H- and ^{13}C-NMR spectra of **9d**

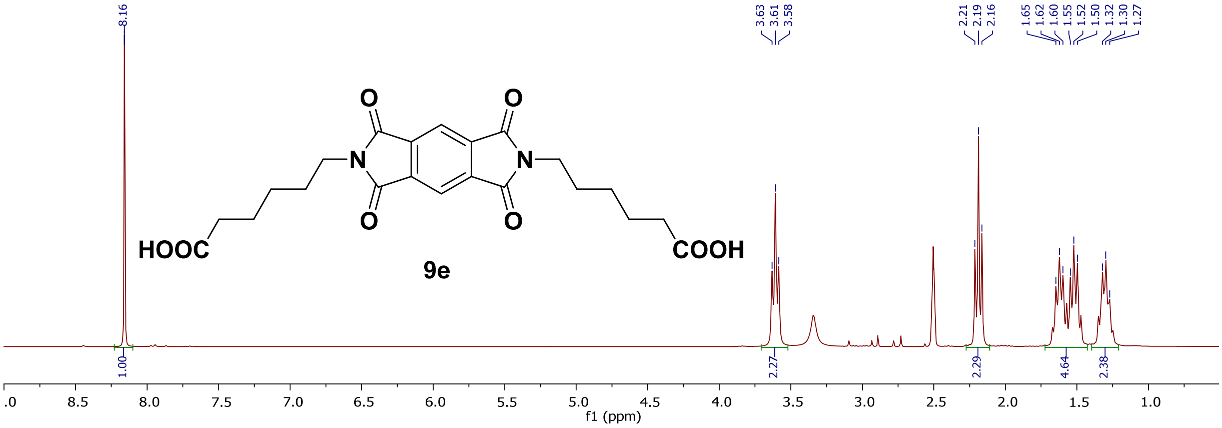

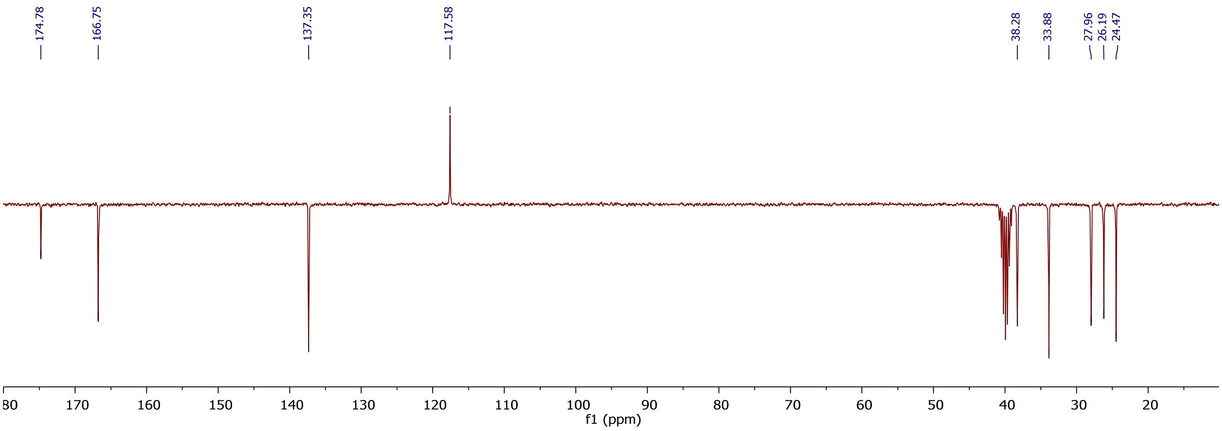

Figure S15 ^{1}H- and ^{13}C-NMR spectra of **9e**
